# Extracellular vesicles from hypoxia-preconditioned microglia promote angiogenesis and repress apoptosis in stroke mice via the TGF-β/Smad2/3 pathway

**DOI:** 10.1038/s41419-021-04363-7

**Published:** 2021-11-09

**Authors:** Lin Zhang, Wei Wei, Xiaoyu Ai, Ertugrul Kilic, Dirk M. Hermann, Vivek Venkataramani, Mathias Bähr, Thorsten R. Doeppner

**Affiliations:** 1 Department of Neurology, University Medical Center Göttingen, Göttingen, Germany; 2 Regenerative and Restorative Medical Research Center, Istanbul Medipol University, Istanbul, Turkey; 3 Department of Neurology, University Hospital Essen, University of Duisburg-Essen, Essen, Germany; 4 Department of Medicine II, University Hospital Frankfurt, Frankfurt, Germany; 5 Institute of Pathology, University Medical Center Göttingen, Göttingen, Germany

**Keywords:** Cellular neuroscience, Neuroimmunology

## Abstract

Systemic transplantation of oxygen−glucose deprivation (OGD)-preconditioned primary microglia enhances neurological recovery in rodent stroke models, albeit the underlying mechanisms have not been sufficiently addressed. Herein, we analyzed whether or not extracellular vesicles (EVs) derived from such microglia are the biological mediators of these observations and which signaling pathways are involved in the process. Exposing bEnd.3 endothelial cells (ECs) and primary cortical neurons to OGD, the impact of EVs from OGD-preconditioned microglia on angiogenesis and neuronal apoptosis by the tube formation assay and TUNEL staining was assessed. Under these conditions, EV treatment stimulated both angiogenesis and tube formation in ECs and repressed neuronal cell injury. Characterizing microglia EVs by means of Western blot analysis and other techniques revealed these EVs to be rich in TGF-β1. The latter turned out to be a key compound for the therapeutic potential of microglia EVs, affecting the Smad2/3 pathway in both ECs and neurons. EV infusion in stroke mice confirmed the aforementioned in vitro results, demonstrating an activation of the TGF-β/Smad2/3 signaling pathway within the ischemic brain. Furthermore, enriched TGF-β1 in EVs secreted from OGD-preconditioned microglia stimulated M2 polarization of residing microglia within the ischemic cerebral environment, which may contribute to a regulation of an early inflammatory response in postischemic hemispheres. These observations are not only interesting from the mechanistic point of view but have an immediate therapeutic implication as well, since stroke mice treated with such EVs displayed a better functional recovery in the behavioral test analyses. Hence, the present findings suggest a new way of action of EVs derived from OGD-preconditioned microglia by regulating the TGF-β/Smad2/3 pathway in order to promote tissue regeneration and neurological recovery in stroke mice.

## Introduction

Cerebral ischemia induces energy depletion, excitotoxicity, and cellular demise [1], resulting in high levels of cytokines and chemokines at the early stage of the disease. The release of such factors further aggravates secondary brain injury due to enhanced excitotoxicity, cytolysis, oxidative stress, and thromboinflammation [2–4]. Several immune competent cells are involved in the process, among which are microglia. The latter are resident immune cells of the brain parenchyma, participating in various signaling pathways of ischemic brain injury [5–8]. Increasing evidence has shown that microglial activation and transition towards M2 polarization play key roles in the inflammatory postischemic response at the early stage of the disease. M2 microglia alter the cellular microenvironment of the ischemic brain by clearing cell debris and by releasing immunomodulatory factors such as galectin-3 and IL-10 [9–12]. Such activated M2 microglia also contribute to axonal growth and cerebral repair for which VEGF, BDNF, IGF-1, and MMP-9 play a pivotal role. The transition of the microglial phenotype, however, depends on the cellular activation state during the course of ischemic brain injury. Balancing a sustained M2 polarization may therefore be a promising anti-inflammatory therapeutic strategy for stroke treatment [12–14].

Microglial M2 transition is stimulated by various cytokines and other appropriate stimuli such as cerebral ischemia [12, 15–17]. The therapeutic potential of microglia is intriguing taking into account previous work, which demonstrates enhanced neurological recovery of rodents treated with oxygen−glucose deprivation (OGD)-preconditioned microglia at an early stroke stage [17]. These microglia display a distinct change of their protein profiles, such as increased levels of the transforming growth factor-β (TGF-β) [17]. Such high levels of TGF-β under early stroke conditions may, in turn, induce anti-inflammatory signaling cascades and an M2 polarization shift within the ischemic brain [17–20]. However, the precise mechanisms as to how TGF-β may affect stroke outcome under such conditions were not studied [17].

Cell-based therapies including microglia transplantation are still hampered due to limited biodistribution of grafted cells and due to cell embolism or tumor formation [21–23]. Besides, cell dosage and transplantation frequency are limited in terms of both safety and costs, generating a need for novel strategies [24, 25]. Hence, extracellular vesicles (EVs), as a cell-free therapy, may overcome these limitations. EVs form a heterogeneous group of vesicles ranging in size from 30 to 1000 nm, which contain non-coding RNAs, DNA, and proteins and which are secreted by virtually all eukaryotic cells [26–28]. Previous studies report that the therapeutic potential of EVs harvested from several stem cell sources is not inferior to their host cells, i.e., EVs are as beneficial as their host cells [29–34]. Indeed, preclinical stroke studies using EVs from non-stem cells hardly exist. The present study therefore analyzes the therapeutic impact of EVs derived from such preconditioned microglia under both in vitro and in vivo stroke conditions. The focus of this approach will lie on microglial M2 transition, TGF-β signaling and on neurological recovery of stroke mice.

## Results

### Oxygen−glucose deprivation induces upregulation of TGF-β1 and stimulates M2 polarization in primary microglia

**Fig. 1:**
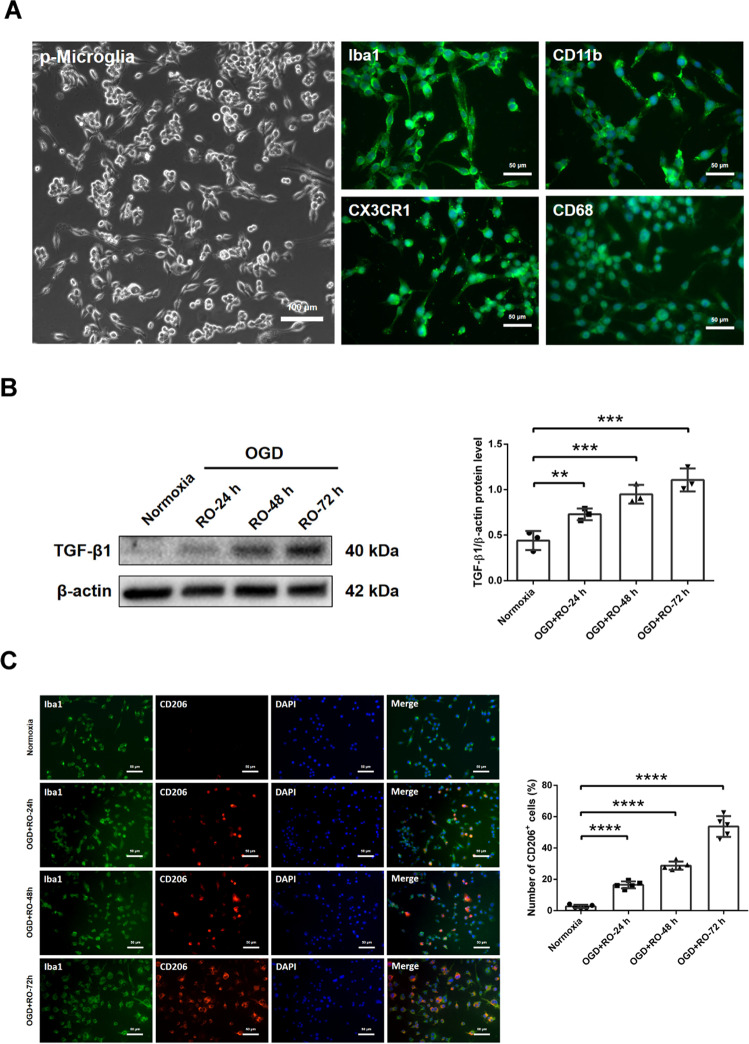
In vitro oxygen−glucose deprivation (OGD) induces upregulation of TGF-β1 and stimulates M2 polarization in primary microglia cells. **A** Phase-contrast images of primary microglia cells under bright-field microscopy and immunofluorescence stainings against the markers Iba1, CD11b, CX3CR1, and CD68 are shown. **B** Quantitative measurement of TGF-β1 protein expression in primary microglia exposed to 4 h of OGD followed by different reoxygenation (RO) periods (24, 48, and 72 h) using Western blot analysis normalized with the housekeeping protein β-actin. Microglia cultivated under standard cell culture conditions (Normoxia) served as control (*n* = 3). **C** Quantitative immunofluorescence staining of Iba1 (green) in primary microglia cells co-localized with CD206 (red), representing M2 polarization of microglia kept under standard cell culture condition or exposed to 4 h of OGD followed by different RO periods (24, 48, and 72 h) (*n* = 5). Data are expressed as mean ± SD, **p < 0.01, ***p < 0.001, ****p < 0.0001. OGD oxygen−glucose deprivation, RO reoxygenation.

Characterization of primary microglia by means of phase-contrast microscopy and immunocytochemistry confirm the microglial cell phenotype as indicated by several specific known markers, that is, Iba1, CD11b, CX3CR1, and CD68 (Fig. 1A). Protein levels of the transforming growth factor beta 1 (TGF-β1) were significantly increased in microglia exposed to 4 h of OGD followed by reoxygenation (RO; Fig. 1B). Interestingly, the increase in protein abundance of TGF-β1 depended on the duration of RO (24, 48, and 72 h), which in turn correlated with the extent of cell injury (Supplementary Fig. I A, B). Immunocytochemistry staining revealed increased patterns of M2 polarization of microglia exposed to 4 h of OGD with different RO times of 24, 48, and 72 h respectively, as indicated by counterstaining against both Iba1 and CD206 when compared to normoxia cell culture conditions (Fig. 1C).

### Isolation and characterization of extracellular vesicles (EVs) from OGD-preconditioned microglia and TGF-β1 knockdown microglia

**Fig. 2:**
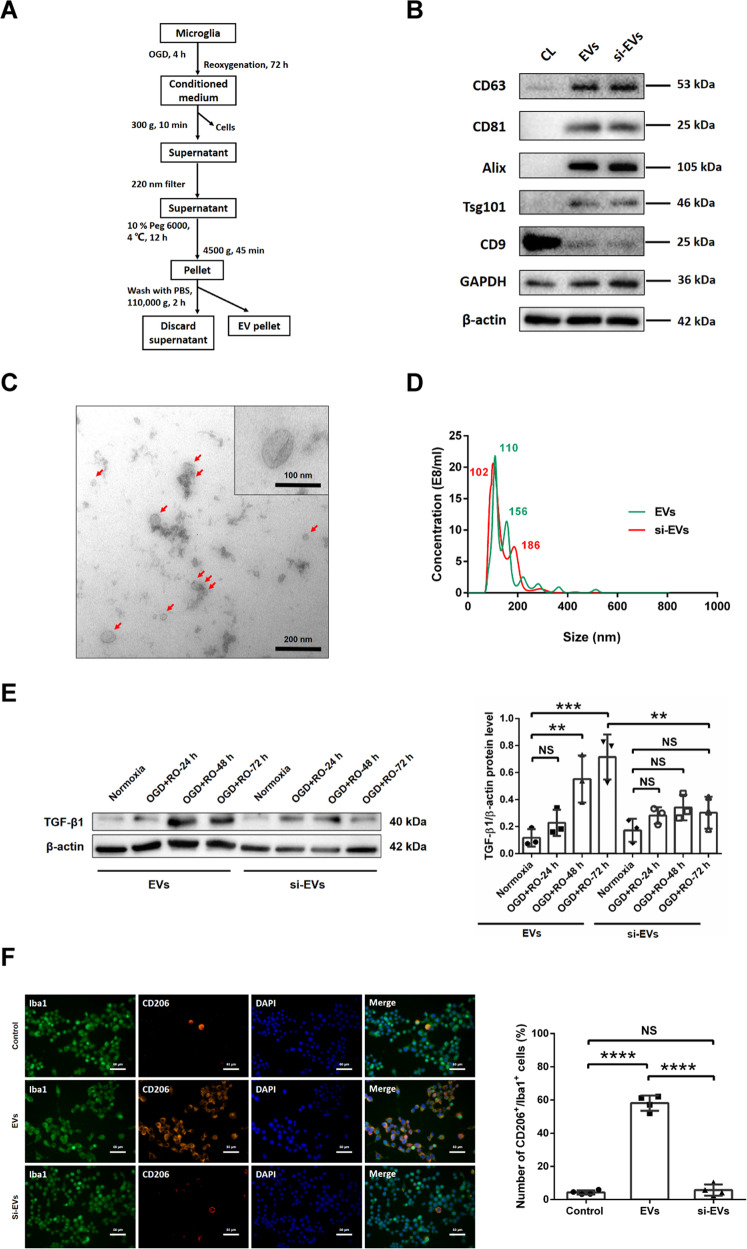
Isolation and characterization of extracellular vesicles (EVs) from OGD-preconditioned microglia and TGF-β1 knockdown microglia. **A** In the schematic diagram, EVs were enriched from the conditioned medium of OGD-preconditioned microglia or TGF-β1 knockdown microglia by the polyethylene glycol (PEG) method. **B** Western blot analysis of EVs against the exosomal markers CD63, CD81, Alix, Tsg101 and CD9 with GAPDH and β-actin serving as loading controls. Western blots were performed on total cell lysates (CL), EV lysates from OGD-preconditioned microglia (EVs) and TGF-β1 siRNA-transfected microglia (si-EVs). **C** Representative transmission electron microscopy (TEM) analysis from EVs enriched by the PEG method. The magnification of cup-shaped EVs is shown on the top right corner. **D** Nanoparticle tracking analysis (NTA) from enriched EVs and si-EVs groups depicting size distribution patterns with peaks at 110 nm (EVs) and 102 nm (si-EVs), respectively. **E** Quantitative analysis of TGF-β1 expression in EVs derived from non-hypoxic microglia or from OGD-preconditioned microglia with different reoxygenation (RO) periods (24, 48, and 72 h) (EV groups), and in EVs derived from TGF-β1 siRNA-transfected microglia with non-hypoxic precondition or with the aforementioned different hypoxic conditions using Western blot analysis normalized with the housekeeping protein β-actin (*n* = 3). **F** Quantitative analysis of M2 phenotype microglia (CD206+/Iba1+) rate by immunofluorescence staining in three groups: PBS vehicle control, aforementioned EV treatment paradigm and si-EV treatment (EVs or si-EVs derived from OGD-preconditioned microglia with 72 h RO). EV incubation promoted M2 polarization of microglia compared with control and si-EV groups (*n* = 4). Data are expressed as mean ± SD. NS no significance, **p < 0.01, ***p < 0.001, ****p < 0.0001. EVs extracellular vesicles, OGD oxygen−glucose deprivation, RO reoxygenation.

Verifying our hypothesis that indeed EVs from preconditioned microglia are the true biological mediators of these cells, EVs were first characterized according to the ISEV guidelines [27]. Primary microglia isolated from the cerebral hemispheres of mouse pups were exposed to OGD as mentioned before. For some experiments, these preconditioned microglia were transfected in order to achieve knockdown of TGF-β1. In our results, around 50% of primary microglia were successfully transfected with 50 nM TGF-β1 siRNA (Supplementary Fig. I C−E). EVs were harvested from the conditioned medium of these microglia (EVs and si-EVs groups) using the PEG method as described in the ‘Materials and methods’ section (Fig. 2A). The subsequent characterization of such enriched EVs included Western blots of EV biomarkers, TEM, and NTA. In the Western blots, enriched EVs from both EVs and si-EVs groups revealed the presence of commonly reported EV biomarkers such as CD63, CD81, Alix, Tsg101, and CD9 compared to the primary microglia cell lysate (non-EV) group (Fig. 2B). TEM analysis showed no significant morphological differences between EVs and si-EVs groups, and representative images with typical cup-like membrane vesicles were displayed in the TEM imaging (Fig. 2C). EVs were further quantified and evaluated for size using NTA, which displayed an EV-like spectrum in both groups. The distribution of different size patterns from the NTA results revealed the majority of EVs to be at about 50−200 nm in size in both groups, and the modes of particle size were 110 and 102 nm, respectively (Fig. 2D). As such, no significant difference between the two enriched EVs from conditioned medium of OGD-preconditioned microglia and OGD-preconditioned TGF-β1 knockdown microglia (EVs and si-EVs groups) was observed.

An analysis of TGF-β1 protein expression revealed an accumulation of this protein in EVs from OGD-preconditioned microglia depending on RO duration. Knockdown experiments resulted in, as expected, significantly reduced levels of TGF-β1 in all conditions (Fig. 2E). According to previous work, TGF-β1 stimulates the microglial transition into an M2 phenotype which contributes to creating an anti-inflammatory extracerebral milieu within the ischemic brain parenchyma [9, 12–14]. Hence, we wondered whether or not increased concentrations of TGF-β1 inside of EVs from preconditioned microglia may enhance the M2 phenotype polarization in cultivated microglia cells, indicating a possible anti-inflammatory positive feedback loop of microglia. Indeed, incubation of cultivated microglia with these EVs resulted in increased M2 polarization (CD206+/Iba1+) of microglia (Fig. 2F), thus supporting our hypothesis.

### EVs derived from OGD-preconditioned microglia promote cell viability, migration and angiogenesis against hypoxic injury in bEnd.3 cells via the TGF-β/Smad2/3 pathway

**Fig. 3:**
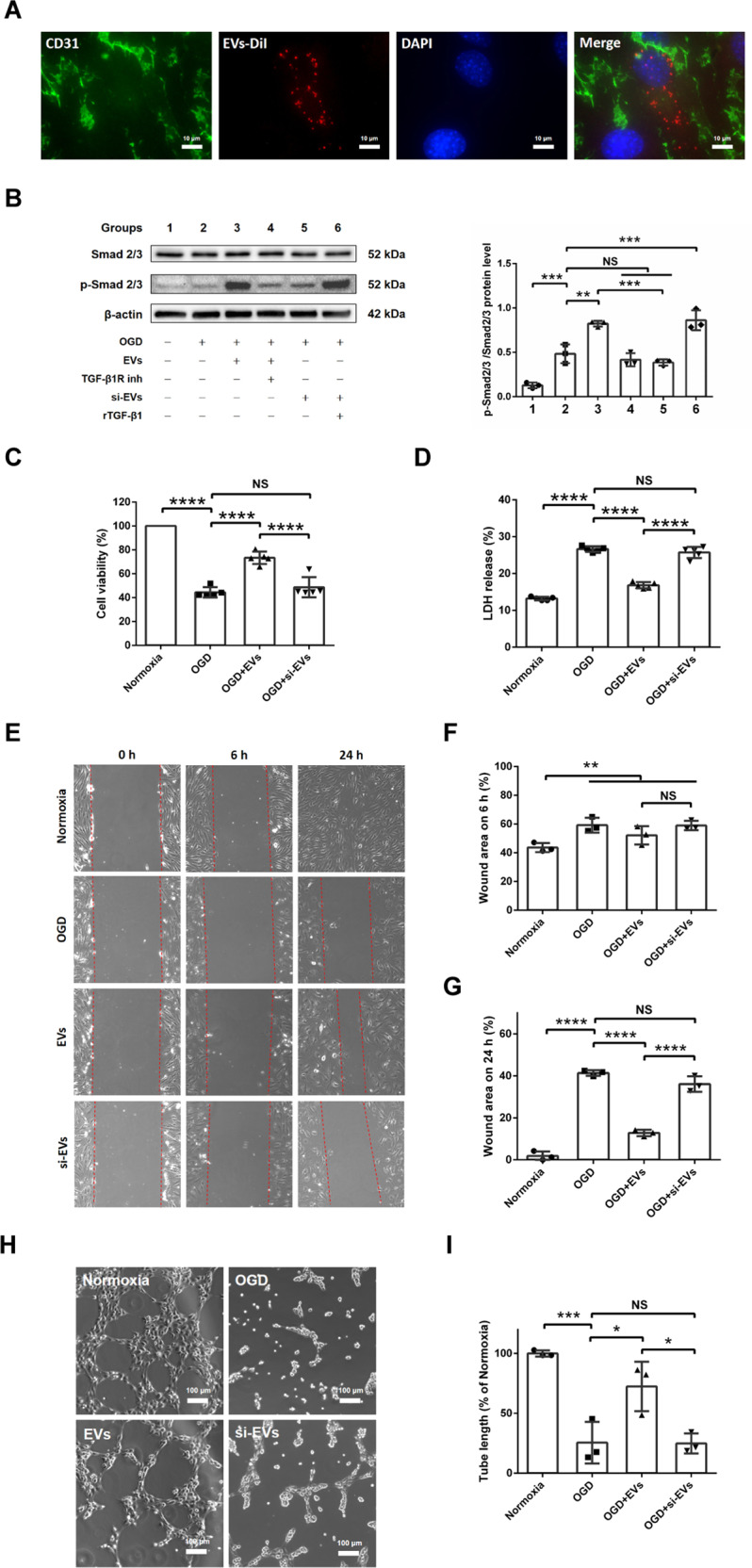
EVs derived from OGD-preconditioned microglia promote cell viability, migration, and angiogenesis in hypoxic bEnd.3 cells via the TGF-β/Smad2/3 pathway. **A** EVs labeled with DiI (red) were taken up into the cytoplasm of CD31+ (green) bEnd.3 endothelial cells. **B** Quantitative analysis of Smad2/3 and p-Smad2/3 expression associated with the TGF-β/Smad2/3 pathway using Western blot analysis normalized with the housekeeping protein β-actin (*n* = 3) in the six groups: group 1 (treatment with drug solvent under normoxic condition), group 2 (OGD/RO treatment with drug solvent), group 3 (1 μg/ml EV incubation during OGD/RO, EVs derived from OGD-preconditioned microglia), group 4 (2 µM TGF-β1 receptor inhibitor treatment in group 3), group 5 (1 μg/ml si-EV incubation during OGD/RO, si-EVs derived from OGD-preconditioned plus TGF-β1 siRNA-transfected microglia), and group 6 (10 ng/ml recombinant TGF-β1 treatment in group 5). **C** Cell viability was analyzed in endothelial cells exposed to 16 h of OGD followed by 24 h of RO using the MTT assay in the four groups: normoxia, OGD control, EV treatment and si-EV treatment during OGD/RO (*n* = 5). Cells incubated in the normoxia control group were defined as 100% cell survival. **D** OGD-induced cell toxicity was further assessed in the lactate dehydrogenase (LDH) release assay (*n* = 5). **E** Representative photos under bright-field microscopy analyzing cell migration after 0, 6 and 24 h after scratch injury in the aforementioned experimental groups. **F**, **G** Quantitative analysis of the scratch assay from (**E**) at 6 h (**F**) and at 24 h (**G**) after the scratch (*n* = 3). **H** Representative photos of the tube formation assay at 6 h after seeding the endothelial cells on the matrigel. **I** Quantitative analysis of tube branch length indicating promoted angiogenesis in hypoxic endothelial cells after the various treatment paradigms (*n* = 3). Data are expressed as mean ± SD. NS no significance, *p < 0.05, **p < 0.01, ***p < 0.001 and ****p < 0.0001. EVs extracellular vesicles, OGD oxygen−glucose deprivation, RO reoxygenation, LDH lactate dehydrogenase.

In order to investigate whether or not microglial-derived EVs act on angiogenesis in vitro, we exposed bEnd.3 endothelial cells to OGD. As shown in Fig. 3A, EVs labeled with DiI (red) were taken up into bEnd.3 endothelial cells (CD31+). Using both immunofluorescence staining and Western blotting analysis, we compared the effect of Dil labeled EVs and naïve EVs. As shown in Supplementary Fig. II A–C, both DiI-EVs and naïve EVs activated the Smad2/3 signaling pathway, and there is no significant difference between them. To confirm that it is indeed TGF-β1 inside such enriched EVs from preconditioned EVs that mediates the biological effects, we designed an extensive in vitro experimental paradigm on bEnd.3 cells exposed to OGD cell injury (Supplementary Fig. II D). Some endothelial cells were incubated with EVs enriched with TGF-β1 (i.e., EVs from preconditioned microglia), whereas other endothelial cells were treated with EVs plus a TGF-β1 receptor inhibitor. Another group of bEnd.3 cells was treated with si-EVs (where TGF-β1 is knocked down) or with si-EVs plus recombinant TGF-β1. Western blot analysis of such treated bEnd.3 cells revealed that treatment with EVs enriched with TGF-β1 activated the TGF-β/Smad2/3 pathway, whereas the p-Smad2/3 protein level after OGD was not upregulated in the EV plus TGF-β1 receptor inhibitor group or the si-EV group (Fig. 3B). Treatment with recombinant TGF-β1, on the contrary, reversed the effects of si-EV treatment on the p-Smad2/3/Smad2/3 ratio.

Further experiments focused on the therapeutic impact of EVs derived from preconditioned microglia against hypoxic injury of bEnd.3 cells. As such, cell viability and cytotoxicity were analyzed by MTT assay and LDH release assay. The reduction of cell viability and elevation of cytotoxicity after 16 h OGD and 24 h RO were reversed by applying EVs from OGD-preconditioned microglia, whereas si-EVs had no beneficial effect in this respect (Fig. 3C, D). Furthermore, the scratch migration assay under OGD conditions demonstrated that EVs enriched with TGF-β1 (i.e., EVs from preconditioned microglia) significantly facilitated bEnd.3 migration during RO by calculating the wound area at 24 h after the scratch when compared to the OGD control and si-EV groups. However, we did not find any significant difference at 6 h after the scratch (Fig. 3E–G). Likewise, tube formation of bEnd.3 cells was significantly impaired when these cells were exposed to OGD and RO. EVs derived from preconditioned microglia reversed this effect, whereas si-EVs had no effect in this respect whatsoever (Fig. 3H, I). Hence, these data are in favor of EVs enriched with TGF-β1 from OGD-preconditioned microglia to promote cell viability, migration and angiogenesis against hypoxic injury in bEnd.3 cells.

### EVs repress neuronal apoptosis in hypoxic cortical neurons via the TGF-β/Smad2/3 pathway

**Fig. 4:**
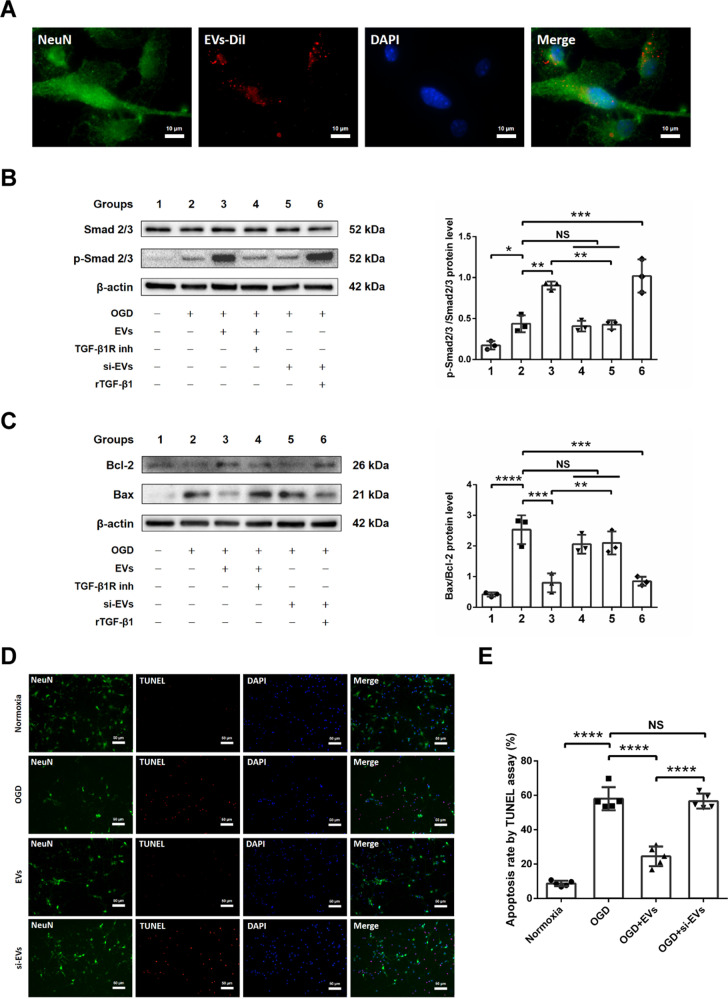
EVs repress neuronal apoptosis in hypoxic cortical neurons via the TGF-β/Smad2/3 pathway. **A** EVs labeled with DiI (red) were taken up into the cytoplasm of NeuN+ (green) primary cortical neurons. **B** Quantitative analysis of Smad2/3 and p-Smad2/3 expression associated with the TGF-β/Smad2/3 pathway using Western blot analysis normalized with the housekeeping protein β-actin (*n* = 3) in the six groups: group 1 (treatment with drug solvent under normoxic condition), group 2 (OGD/RO treatment with drug solvent), group 3 (1 μg/ml EV incubation during OGD/RO, EVs derived from OGD-preconditioned microglia), group 4 (2 µM TGF-β1 receptor inhibitor treatment in group 3), group 5 (1 μg/ml si-EV incubation during OGD/RO, si-EVs derived from OGD-preconditioned plus TGF-β1 siRNA-transfected microglia), and group 6 (10 ng/ml recombinant TGF-β1 treatment in group 5). **C** Quantitative analysis of anti-apoptotic protein Bcl-2 and pro-apoptotic protein Bax expression using Western blot analysis normalized with the housekeeping protein β-actin in the same six groups (*n* = 3). **D**, **E** Quantitative analysis of apoptotic cell (red) rate in primary cortical neurons (NeuN, green) by TUNEL staining (red) in the four groups: normoxia, OGD control, EV treatment and si-EV treatment during OGD and RO (*n* = 5). Data are expressed as mean ± SD. *p  <  0.05, **p < 0.01, ***p < 0.001, and ****p < 0.0001. EVs extracellular vesicles, OGD oxygen−glucose deprivation, RO reoxygenation, Bcl-2 B-cell lymphoma 2 protein, Bax Bcl-2-associated X protein.

Likewise, we analyzed the therapeutic impact of EVs on primary cortical neurons exposed to OGD. As shown for endothelial cells (Fig. 3A), EVs labeled with DiI (red) are taken up by primary cortical neurons (Fig. 4A). To determine whether EVs from OGD-preconditioned microglia affect neurons against hypoxic injury via the TGF-β/Smad2/3 pathway, we applied the same experimental design as described for the endothelial bEnd.3 cells. As such, TGF-β blocking abolished the effect of EV treatment on p-Smad2/3. Recombinant TGF-β1 activated the TGF-β/Smad2/3 pathway compared to si-EV treatment alone, similar to the treatment with TGF-β enriched EVs (Fig. 4B). Furthermore, we investigated the expression of the pro-apoptotic protein Bax and the anti-apoptotic protein Bcl-2 in hypoxic neurons of the aforementioned six experimental groups (Fig. 4C). EVs decreased neuronal apoptotic signaling in hypoxic neurons via the TGF-β/Smad2/3 pathway, whereas application of the TGF-β1 receptor inhibitor or si-EVs reversed this effect. Assessment of cell injury by means of TUNEL staining confirmed the Western blot results, i.e., EVs from OGD-preconditioned microglia but not si-EVs significantly reduced apoptotic cell injury (Fig. 4D, E). The in vitro experimental paradigm on bEnd.3 cells and cortical neurons were stated in Supplementary Fig. II E.

### Delivery of EVs from OGD-preconditioned microglia induces the TGF-β/Smad2/3 signaling pathway in ischemic mouse hemispheres

**Fig. 5:**
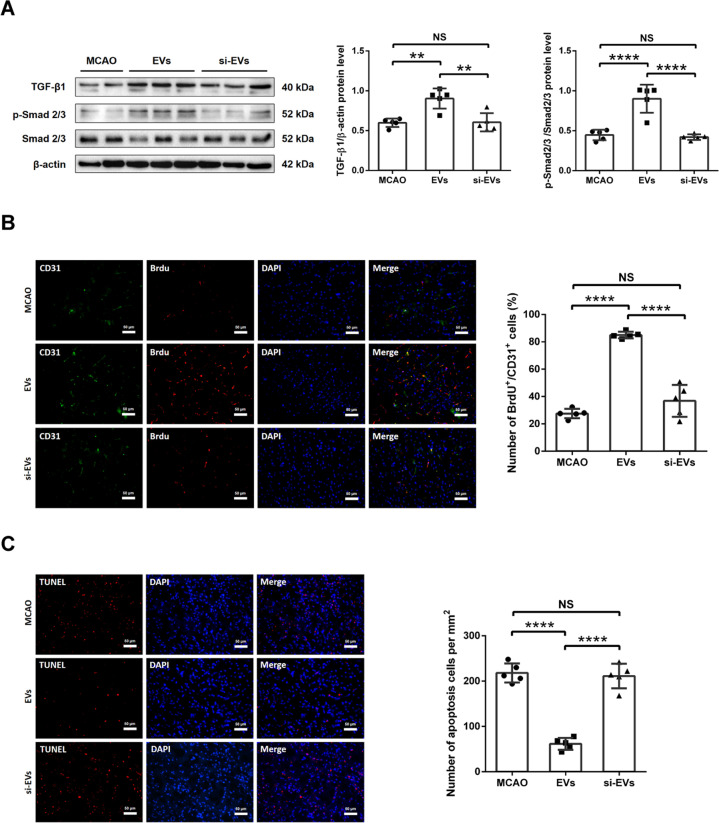
EV administration induces angiogenesis and diminishes neuronal apoptosis in the mouse middle cerebral artery occlusion (MCAO) stroke model. After exposing to 60 min of MCAO, mice were treated with PBS (MCAO control) or intravenously treated with EV administration at the beginning of the reperfusion and at 6 h later with another EV administration. **A** Quantitative analysis of TGF-β1, p-Smad2/3 and Smad2/3 expression in MCAO mice, MCAO mice treated with EVs and MCAO mice treated with si-EVs by Western blot analysis of the ischemic hemisphere. Western blot was normalized with the housekeeping protein β-actin (*n* = 5). **B** Quantitative analysis of proliferating cell (BrdU, red) rate in endothelial cells (CD31, green) of the ischemic striatum at post-MCAO day 7 by immunofluorescence staining in the aforementioned groups. EV administration increases the number of proliferating endothelial cells (BrdU+/CD31+) in the ischemic striatum compared with the si-EV group (*n* = 5). **C** Quantitative analysis of apoptotic cell (red) per mm^2^ in the ischemic striatum at post-MCAO day 7 by TUNEL staining in the same groups (*n* = 5). Data are expressed as mean ± SD. **p < 0.01, ***p < 0.001, and ****p < 0.0001. EVs extracellular vesicles, MCAO middle cerebral artery occlusion, PBS phosphate-buffered saline, BrdU 5-bromo-2ʹ-deoxyuridine, TUNEL terminal deoxynucleotidyl transferase dUTP nick end labeling.

To analyze the role of EVs from OGD-preconditioned microglia on the regulation of the TGF-β/Smad2/3 pathway in ischemic stroke, we studied TGF-β, p-Smad2/3 and Smad2/3 protein expression in mice submitted to 60 min of MCAO. Western blots of the samples from ischemic hemispheres revealed that both the TGF-β protein levels and the expression ratios of p-Smad2/3/Smad2/3 were increased in the EV treatment group, whereas no significant differences were observed in the si-EV administration group (Fig. 5A). These results suggest that the delivery of EVs from OGD-preconditioned microglia activates the TGF-β1-dependent Smad2/3 pathway in ischemic hemispheres of stroke mice.

### EV administration induces angiogenesis and diminishes cell injury in ischemic mouse hemispheres

In accordance with the aforementioned in vitro findings, we further hypothesized that delivery of EVs from OGD-preconditioned microglia may stimulate angiogenesis and abolish apoptosis in the ischemic brain. A co-expression analysis of the cell proliferation marker BrdU and the endothelial cell marker CD31 revealed increased levels of BrdU+/CD31+ cells on post-ischemia day 7 within the ischemic striatum of animals treated with EVs, a finding which was not confirmed in mice treated with si-EVs (Fig. 5B). Likewise, EV administration significantly reduced ischemic cell death rates, whereas the number of TUNEL + apoptotic cells per mm^2^ was not affected by the treatment of si-EVs from TGF-β1 knockdown microglia (Fig. 5C).

### Delivery of EVs with enriched TGF-β1 augments M2 polarization of microglia in stroke mice

**Fig. 6:**
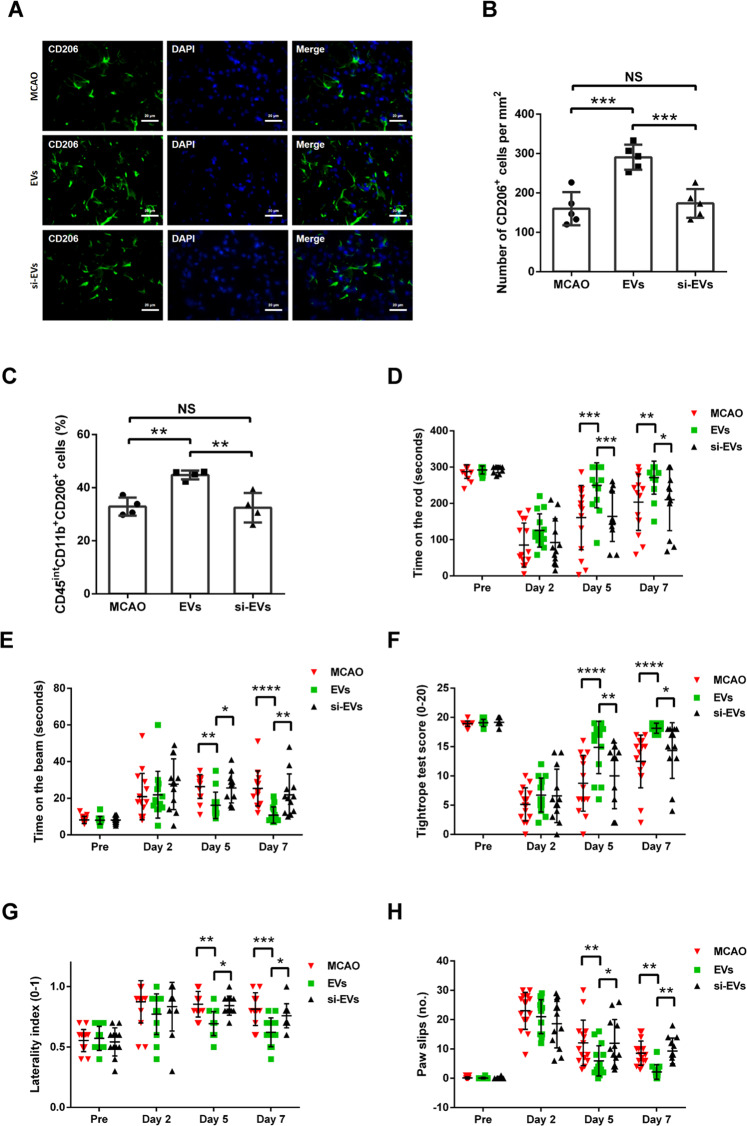
Delivery of TGF-β1-enriched EVs augments M2 polarization of microglia and mitigates postischemic motor coordination impairment in stroke mice. **A**, **B** Quantitative analysis of M2 polarization of microglia cells (CD206, green) in the ischemic striatum at post-MCAO day 7 by immunofluorescence staining in the three groups: MCAO control, MCAO with EV treatment, and MCAO with si-EV treatment. EV infusion increases M2 polarization rates of resident microglial cells in the ischemic striatum compared with the si-EVs group (*n* = 5). **C** At post-MCAO day 7, flow cytometry of ischemic hemispheres showing a significant increase of M2 microglial cells (CD45^int^CD11b^+^CD206^+^) in the EV treatment group compared to the MCAO control and the MCAO si-EV treatment group (*n* = 4). Delivery of EVs reduces postischemic motor coordination impairment. Motor coordination was evaluated using the rotarod test (**D**), balance beam test (**E**), tightrope test (**F**), corner turn test (**G**), and paw slips recording (**H**) 1 day before stroke (pre) as well as 2, 5 and 7 days after stroke. All animals were accordingly trained before the induction of stroke in order to ensure proper test performance (*n* = 5). Data are expressed as mean ± SD. NS no significance, *p < 0.05, **p < 0.01, and ***p < 0.001. EVs extracellular vesicles, MCAO middle cerebral artery occlusion.

The cellular immune response within the ischemic brain is a key element in the pathophysiology of stroke, which is closely linked to M2 phenotype transition of residing microglia. Since EVs from OGD-preconditioned microglia induce angiogenesis and anti-apoptosis (Fig. 5), we wondered whether or not EVs with enriched TGF-β1 have an impact on early M2 polarization of microglia in the postischemic brain. Therefore, the brain tissue of the ischemic hemisphere was analyzed by immunohistochemistry staining and flow cytometry on day 7 after MCAO. We found a significantly higher ratio of the M2 phenotype of microglia (CD206+) in the EV treatment group in comparison to the MCAO control group. On the contrary, delivery of si-EVs from TGF-β1 knockdown microglia had no effect on the M2 polarization of microglia in ischemic hemispheres (Fig. 6A–C). Representative flow cytometry measurement in above groups were shown in Supplementary Fig. III C.

### Delivery of EVs with enriched TGF-β1 mitigates postischemic motor coordination impairment in stroke mice

In light of the aforementioned in vitro data on endothelial cells and cortical neurons, we investigated whether EVs from OGD-preconditioned microglia improve neurological recovery after cerebral ischemia in mice. Delivery of these EVs resulted in significantly better test performance of the animals in the rotarod test, balance beam test, tightrope test, corner turn test as well as in the recording of paw slips on days 5 and 7 when compared with MCAO control groups and si-EV treatment groups. However, these beneficial effects were not immediately visible on day 2 after stroke induction, indicating a time-dependent process for EV-induced neuroregeneration (Fig. 6D–H). Taken together, the behavior test results indicate that EV treatment significantly prevents postischemic motor coordination impairment in stroke mice.

## Discussion

Using both an in vitro and an in vivo stroke model, the present study analyzed the therapeutic value of EVs derived from preconditioned microglia with regard to angiogenesis, anti-apoptosis, and immunomodulation. Our results suggest that OGD-preconditioning transfers microglia into the anti-inflammatory M2 phenotype, which yields enrichment of the TGF-β1 protein inside of EVs harvested from such microglia. These TGF-β1-enriched EVs cross the blood−brain barrier and reach the injured brain parenchyma, resulting in increased numbers of resident anti-inflammatory M2 microglia within the ischemic brain. On the signaling level, TGF-β1 activates the Smad2/3 signaling pathway, ultimately contributing to cellular protection and increased neurological recovery both in vitro and in vivo. Interfering with this signaling cascade, however, undermines the beneficial effect of preconditioned EVs as observed in the TGF-β1 receptor inhibitor or the si-EV experiments.

Microglia play a crucial role in physiological brain function and are also involved in neuroprotection against stroke via facilitating neurogenesis, angiogenesis, synaptic remodeling, and intercellular dynamic crosstalk [5, 6, 12, 35–39]. Although a strict differentiation between M1 and M2 microglia is certainly oversimplified, M1 microglia are generally considered to be pro-inflammatory, whereas anti-inflammatory M2 microglia are beneficial in terms of tissue recovery after stroke [5, 12, 13, 15, 18, 36, 40–42]. As a matter of fact, the temporal resolution of M2 microglia under ischemic brain conditions ranges from 12 h to several days poststroke, with a maximum between 1 and 3 days [9, 11]. Hence, shifting the balance towards the M2 restorative phenotype at this early time course of stroke may inherit a feasible and attractive therapeutic target. Indeed, intravascular administration of beneficial microglia improves the functional outcome after ischemic stroke [17, 40, 43]. Cell-based therapies using M2 microglia, however, do not only rely on the clearing abilities of these grafted cells, but also on their secretion profile of various protective remodeling factors and cytokines like VEGF, MMP-9, and TGF-β1 [12, 17, 40]. Since a great deal of eukaryotic cells affect target cells––at least in part––by secreting EVs [44–46], it stands to reason that the published effects of OGD-preconditioned microglia are due to EVs rather than to microglia themselves [17].

Several preclinical studies in various neurological disease models observed neuroprotective effects due to a cell-based therapy, which was attributed to therapeutic factors released by these grafted cells [47]. Of note, our present study demonstrates that cell-derived EVs may actually be this factor. EVs derived from various cell sources can pass the blood−brain barrier and induce neuroprotection and neuroregeneration under stroke conditions [26–28]. Interestingly, EVs are a relatively novel tool known for delivering functional cargo to modulate downstream pathways in target cells. Due to their lack of immunogenicity and little side effects in comparison to stem cell transplantation, this feature makes EVs promising candidates for gene and protein delivery [44, 45]. As a matter of fact, we found EVs from OGD-preconditioned microglia to not only successfully cross the blood−brain barrier, but also to be high in TGF-β1 concentration. The latter is likely to be the key compound of EVs derived from such OGD-preconditioned microglia.

As a remodeling factor from microglia, numerous studies suggest TGF-β1 which is associated with the TGF-β/Smad2/3 signaling pathway may exert a protective effect on angiogenesis, axonal outgrowth, anti-apoptosis, and immunomodulation in ischemic stroke [19, 48–52]. The activation of the downstream cascades thus regulates the transcription of key target genes involved in the control of the cell cycle and the extracellular matrix metabolism [53, 54]. In animal stroke models, depletion of TGF-β1 has been shown to increase inflammatory responses and deteriorate microglial CNS homeostatic function [55], whereas delivery of TGF-β1 protein via intravascular or intranasal delivery routes attenuated poststroke neuroinflammation and improved the functional outcome [20, 49, 56–58]. Our results are consistent with previous studies on the role of TGF-β1, as incubation with EVs derived from OGD-preconditioned microglia activated TGF-β/Smad2/3 signaling pathway in both endothelial cells and cortical neurons. The importance of this pathway is further underlined by the observation that the therapeutic effect of EVs from preconditioned microglia is significantly hampered when the TGF-β1 receptor is blocked or TGF-β1 is knocked down in cultivated microglia before EV harvesting. When the TGF-β1 pathway is not interrupted, EVs from preconditioned microglia prove to be a powerful tool for shifting the balance of resident microglia towards the M2 phenotype, resulting in increased neurological recovery and enhanced neuroregeneration of the ischemic brain.

In conclusion, the present work demonstrates EVs derived from OGD-preconditioned microglia to promote neuroprotection and neurological recovery in a TGF-β1-dependent manner, for the first time. This novel work underlines the therapeutic potential of EVs under stroke conditions. Nevertheless, the specific downstream signaling pathways displayed herein need further characterization, thus making additional studies necessary before a clinical translation is in order.

## Materials and methods

### Cell cultures

Cells were tested for potential mycoplasma contamination before the actual start of the experiments. Mouse brain endothelial cells (bEnd.3, CRL-2299™, American Type Culture Collection, Manassas, Virginia, USA) were seeded in TC-plates (Sarstedt, Nuembrecht, Germany) and cultured under confluent conditions at a density of 60,000 cells/cm^2^. Cells were cultured with 10% fetal bovine serum (FBS)-containing medium (Dulbecco’s modified Eagle medium/Ham’s F12, Biochrom GmbH, Berlin, Germany).

Primary mouse brain microglia were prepared using a protocol based on the method of Hong et al. [59]. Three C57BL/6J male mouse pups at postnatal days 0−2 were decapitated, and whole brains were removed and placed in HBSS on ice. From each brain, both the cerebellum and the olfactory bulbs were removed with a sterile blade. The meninges were dissected from the cortex hemispheres by pulling with forceps. Pooled cortical tissue was digested with 0.25% trypsin at 37 °C for 15 min. A volume of 200 µl of 10 mg/ml DNase was added to digest the sticky DNA released from dead cells. The cell suspension was centrifuged at 300 × *g* for 5 min, and the pellet was gently suspended with 5 ml warm culture media using a 1 ml pipet tip. The homogenous cell suspension was transferred to a 15 ml tube and centrifuged at 300 × *g* for 5 min. The resulting pellet was suspended in 20 ml of astrocyte full medium (DMEM supplemented with 10% FBS and 1% penicillin/streptomycin) and cultivated on Poly-d-lysine (PDL)-coated T75 flasks. The culture medium was changed the next day to remove non-adherent cell debris. The culture medium was changed every 5 days, thereafter. After 5−7 days, astrocytes at the bottom of the flask would form a confluent cell layer, and microglia would grow on top of the astrocytic layer. To collect microglia, purified microglia were collected in conditioned culture medium after vigorously taping the flasks on the bench top. A hemocytometer was used to count the floating cell density and the cells were seeded at 50,000 cells/cm^2^ in PDL-coated culture dishes or staining slides for the upcoming experiments.

For the preparation of primary cortical neurons, pregnant C57BL/6J mice were sacrificed by CO_2_ inhalation at embryonic day 16.5. Embryos were dissected, and tissue pieces were trypsinized and dissociated using a fire-polished Pasteur pipette. Cells were seeded on poly-l-ornithine/laminin (Sigma-Aldrich, St. Louis, MO, USA)-coated 6-well or 24-well plates at a density of 100,000 cells/cm^2^ containing neurobasal medium (Gibco, Darmstadt, Germany) with additional transferrin (Sigma-Aldrich, St. Louis, MO, USA), penicillin/streptomycin (PS; Gibco, Darmstadt, Germany), l-glutamine (Seromed, Dollnstein, Germany), and B27 supplement (Gibco, Darmstadt, Germany). Cells were used for subsequent experiments after 4 days of cell culture.

### Oxygen−glucose deprivation

The cells were exposed to OGD when they reached 80−90% confluence. For OGD, the cells were incubated in glucose-free balanced salt solution (BSS0) solution (116 mM NaCl, 5.4 mM KCl, 0.8 mM MgSO_4_, 1 mM NaH_2_PO_4_H_2_O, 26.2 mM NaHCO_3_, 10 mM HEPES, 0.01 mM glycine and 1.8 mM CaCl_2_, pH 7.2−7.4) and transferred to a hypoxia incubator chamber containing 0.2% O_2_, 5% CO_2_, and 70% humidity (Toepffer Lab Systems, Goeppingen, Germany). For reoxygenation (RO) after having removed the BSS0 solution, the cells were incubated in cell culture medium for 24 h in the 5% CO_2_ incubator at 37 °C (standard cell culture condition). Thereafter, the cells were treated for the next experiments. According to the different cell types with specific tolerance to hypoxia injury, primary microglia, bEnd.3 and cortical neuron were exposed to OGD for 4, 16 and 6 h, respectively. For the in vitro experiments, EV treatment was performed during both OGD and RO periods.

### TGF-β1 siRNA transfection of microglia

Primary microglia were stably transfected with FlexiTube small interfering RNA (siRNA) for mouse TGF-β1 (NM_011577) or negative control siRNA using HiPerFect Transfection Reagent (Qiagen, Hilden, Germany). The siRNA transfection was performed in serum-free medium, following the manufacturer’s instruction. The most efficient target sequence for RNA interference was selected from the sequence provided by Qiagen (#SI00201684). All siRNAs were tested for mRNA knockdowns by real-time polymerase chain reaction (PCR). After 24 h of transfection, the cells were used for subsequent experiments. More details about the TGF-β1 mRNA expression after treatment with different concentrations of TGF-β1 siRNA by using qRT-PCR can be found in Supplementary Fig. I C.

### Quantitative real-time polymerase chain reaction (qRT-PCR)

The microglial cells were analyzed to quantify gene expression after transfection. Total RNA was extracted using TRIzol reagent (Invitrogen, Waltham, Massachusetts, USA) according to the manufacturer’s instructions. The KAPA SYBR^®^ FAST One-Step Kit for LightCycler^®^480 (Merck Group, Darmstadt, Germany) was used to perform qRT-PCR according to the manufacturer’s instructions, and the TGF-β1 primers (forward 5′-CCTGTCCAAACTAAGGC-3′ and reverse 5′-GGTTTTCTCATAGATGGCG-3′) were purchased from Eurofins Genomics (Ebersberg, Germany). The relative quantity levels were calculated with the 2^−ΔΔCt^ method using β-actin (forward 5′-CGTGCGTGACATCAAAGAGA-3′ and reverse 5′- CCCAAGAAGGAAGGCTGGA-3′) as the interval standard control. The reported results are based on three independent experiments on separate batches of cells.

### EV enrichment from OGD-preconditioned microglia

Primary microglia were cultured to 80−90% confluence in complete culture medium (DMEM containing 10% fetal bovine serum and 1% penicillin/streptomycin). After 4 h of OGD treatment, the microglia were incubated in complete medium according to different reoxygenation time (24, 48, and 72 h). Then, the complete medium was replaced with serum-free medium. Twenty-four hours later, the medium was collected, and living cells and larger vesicles were removed by centrifugations at 300 × *g* for 10 min. The samples were filtered through 0.22 µm pore filters (TPP Techno Plastic Products AG, Trasadingen, Switzerland).

EVs were enriched from the conditioned medium using the polyethylene glycol (PEG) precipitation method, as previously described [20, 31]. In brief, PEG precipitation was performed at a final concentration of 10% PEG 6000 (50% wt/vol; Merck Group, Darmstadt, Germany) and 75 mM NaCl. After incubation for 12 h at 4 °C, the EVs were concentrated by centrifugation for 45 min at 4500 × *g*. EV pellets were dissolved in PBS and precipitated by ultracentrifugation for 2 h at 110,000 × *g* (Optima XPN-80 Ultracentrifuge, Beckman Coulter, Brea, California, USA) (Fig. 2A). The pellets were resuspended in 500 µl aliquots of PBS. In addition, by using iodixanol gradient centrifugation, we enriched EVs from the middle fractions, whereas non-EV nanoparticles and soluble proteins located in the bottom fractions were collected and removed [60]. The final EV pellets were resuspended in PBS and stored at −80 °C for further experiments.

### Characterization and purification of EVs

Details about transmission electron microscopy (TEM), nanoparticle tracking analysis (NTA) and Western blotting of EV markers can be found in the Supplementary Materials. Importantly, the amount of EVs applied to cells was calculated by the following process. The EVs from 720 ml conditioned medium were diluted in 500 µl of PBS at a concentration 25.2 µg/µl. In this perspective, these EVs were derived from 2.1 × 10^8^ cell equivalents. In our previous experiments [60], several different EV concentrations (0.1, 1 and 10 µg/ml of the cell culture medium or BSS0 solution) were chosen in order to define the optimal EV dosage of the experiment. For the optimal EV concentration in vitro, we suggested EVs to be diluted to 1 µg/ml in cell culture medium or BSS0 solution. The application solution contained 1 µg EVs from 1.67 × 10^4^ cell equivalents per milliliter of cell culture medium or BSS0 solution. This refers to a total amount of 1.1 × 10^8^ particles, as measured by means of NTA (see Supplementary Information). When fluorescent EVs were needed, the supernatant was incubated with 10 µM DiI, a lipophilic membrane dye, for 1 h at 37 °C in the dark. Then, DiI-labeled EVs were collected by ultracentrifugation at 110,000 × *g* for 2 h at 4 °C. Pellets were washed with PBS to remove excess dye, and final pellets were suspended in PBS. In addition, we applied the iodixanol gradient centrifugation to separate DiI-labeled EVs in middle fractions from the excess DiI dye in the light fractions as well as protein aggregates lacking EVs in the dense fractions.

### Drug treatment in bEnd.3 cells and primary cortical neurons

After seeding, the medium of bEnd.3 was refreshed next day and incubated for another 24 h. Likewise, the medium of primary cortical neurons was half replaced next day and incubated for another 3 days before the OGD treatment. For the drug treatment, the TGF-β1 receptor (TGF-β1R) inhibitor (SB-525334; Sigma-Aldrich, Germany) and the recombinant mouse TGF-β1 (#763104; BioLegend, USA) were added to the BSS0 solution or culture medium during the OGD and 24 h RO period. Control samples were given equivalent volumes of vehicle PBS. Thus, the cells including bEnd.3 and primary cortical neurons were divided into the following six groups: group 1 (treatment with drug solvent under normoxic condition), group 2 (OGD/RO treatment with drug solvent), group 3 (1 μg/ml EV incubation during OGD/RO, EVs derived from OGD-preconditioned microglia), group 4 (2 µM TGF-β1 receptor inhibitor treatment in group 3), group 5 (1 μg/ml si-EV incubation during OGD/RO, si-EVs derived from OGD-preconditioned and TGF-β1 siRNA-transfected microglia), and group 6 (10 ng/ml recombinant TGF-β1 treatment in group 5).

### Cell viability and cytotoxicity assays

Cell viability was measured by a colorimetric assay by using the MTT (Thiazolyl Blue Tetrazolium Bromide, Sigma-Aldrich, St. Louis, MO, USA) viability assay according to the protocol [61]. Following the OGD and RO, cell viability data were presented as relative changes in percent compared to untreated controls. Absorbance was measured at 450 nm using a microplate reader (Tecan Sunrise^TM^, Austria). Furthermore, cytotoxicity was determined by a detection assay that measured the release of lactate dehydrogenase (LDH) from cells to reflect levels of cytotoxicity. A 50 µl aliquot of medium from the samples was transferred to another new 96-well plate. The equivalent dose of manufacturer-provided test reagent was added to each well for measurement of the release of LDH from the cells. Optical density was determined at 490 nm using a microplate reader.

### Scratch migration assay

bEnd.3 cells were seeded in six-well dishes at a density of 60,000 cells/cm^2^ and treated with vehicle PBS, EVs or si-EVs. After 16 h of OGD treatment, a straight scratch in the cell monolayer was made using a 10 µl sterile pipette tip. The cells were washed twice and incubated in fresh cell culture medium supplemented with 10% serum with the following treatment: vehicle PBS, EVs or si-EVs. The images from at least four random areas were captured using an inverted microscope (Eclipse Ts2R; Nikon, Japan) at 6 and 24 h for each test condition after scratching. The rate of wound healing was estimated by measuring the area between the borders of the wound. Each experiment was repeated at least three times.

### Matrigel tube formation

The tube formation assay was performed according to the method described by DeCicco-Skinner et al. [62]. After 24 h of reoxygenation, the groups were starved overnight including the normoxia group using cell culture medium supplemented with 0.2% serum before performing the tube formation assay. The 24-well plates were coated with 250 µl of matrigel (10 mg/ml; #354230, Corning, New York, USA) and incubated at 37 °C for 30 min to promote jelling. A number of 100,000 bEnd.3 cells was resuspended in 300 µl growth medium and added to each well with 1 µg/ml EVs, 1 µg/ml si-EVs and the same volume of vehicle PBS. After incubation in the CO_2_ incubator at 37 °C for 6 h, images from at least four random areas were captured using an inverted microscope (Eclipse Ts2R; Nikon, Japan) in each group. The tube branch length was measured by software ImageJ (National Institutes of Health, USA) with the “Angiogenesis Analyzer” plugin. Each experiment was repeated at least three times.

### Middle cerebral artery occlusion (MCAO) and EV administration

All animal studies were performed with governmental approval according to the NIH guidelines for the care and use of laboratory animals. Animals were strictly and equally randomized to the various groups, and experimenters were blinded from the treatment paradigm and where not involved in data analysis. The survival rates of mice in each group were recorded in Supplementary Table 1. The induction of transient focal cerebral ischemia in male C57BL/6J mice aged 10 weeks (Janvier Labs, Le Genest-Saint-Isle, France) was obtained using the MCA occlusion model as previously described [63]. Only male mice were studied in order to avoid the interference of the hormonal disturbances of female mice after MCAO surgery. In short, under anesthesia with 2% isoflurane and 0.8 l/min O_2_, the right common carotid artery was isolated and a 6-0 nylon silicon-coated monofilament (Doccol Corporation, Massachusetts, USA) inserted. The filament was gently pushed forward towards the MCA where it was placed for 60 min. After filament removal, the wounds were carefully sutured. The occlusion and reperfusion were monitored under constant laser doppler flow (LDF). The mice were exposed to MCAO followed by administration of normal saline and EVs (10 µg in 200 µl PBS) via femoral veins at the onset of reperfusion and 6 h post-MCAO. Each mouse was given 10 µg of EVs each time which were derived from 1.67 × 10^5^ cell equivalents, referring to a total particle number of 1.1 × 10^9^. This optimal EV concentration was chosen in vivo according to our previous studies [60]. All mice were sacrificed at 7 days post-MCAO, and brain samples were prepared for the Western blotting and immunofluorescence staining. The in vivo experimental paradigm in stroke mice were stated in Supplementary Fig. III A.

### Western blotting

Brain samples and cell samples were lysed in a buffer containing 50 mM Tris, 1% Triton-X 100, 131 mM sodium chloride, 1 mM sodium diphosphate, 1 mM sodium fluoride, 1 mM EDTA, 1% protease inhibitor, and 1% phosphatase inhibitor with a homogenisator for 10 min and subsequently centrifuged at 4 °C with 16,000 rpm for 10 min. The supernatant was collected and quantification of the protein concentration photometrically accomplished (Pierce^TM^ BCA Protein Assay Kit, Thermo Fisher Scientific, Waltham, Massachusetts, USA). Reducing sample buffer (Carl Roth, Karlsruhe, Germany) was added, and the samples were heated for 5 min at 95 °C. Thereafter, equal amounts of proteins were separated on 8−12% SDS-PAGE and transferred onto nitrocellulose membrane (Bio-Rad, Hercules, California, USA). Following transfer, the membranes were blocked for 1 h and incubated with the primary antibodies TGF-β1, Smad2/3, p-Smad2/3, Bax, Bcl-2 and β-actin overnight. After washing with tris-buffered saline supplemented with 0.1% Tween® 20 detergent (TBS-T) three times, the blots were incubated with a horseradish-peroxidase-coupled secondary anti-mouse-antibody or anti-rabbit-antibody (1:10,000) for 1 h. The specific antibody working dilutions are given in the resources table (Supplementary Table 2). The membranes were bathed in ECL reagent and developed with imaging system ChemiDoc^TM^ XRS+ (Bio-Rad). The full scans of the Western blots were presented in the Supplementary Materials.

### Immunohistochemistry and immunocytochemistry staining

Brain samples from C57BL/6J mice after perfusion were fixed in 4% paraformaldehyde overnight, dehydrated with 30% sucrose and prepared in 16 μm cryostat sections. The brain sections were blocked with buffer containing 2% BSA (bovine serum albumin), 10% DS (donkey serum), 0.25% Triton X-100 in phosphate-buffered saline supplemented with 0.05% Tween® 20 detergent (PBS-T). The sections were incubated overnight with the following primary antibodies: NeuN, Iba1, CD31, BrdU, and CD206. Thereafter, the sections were incubated for 1 h with the following appropriate Cy-3-labeled or Alexa Fluor 488-labeled secondary antibodies (1:10,000, Jackson ImmunoResearch, Ely, UK) followed by 4ʹ,6-Diamidin-2-phenylindol (DAPI, 1:10,000; AppliChem, Darmstadt, Germany) staining. For cells, the slides or wells were fixed with 4% paraformaldehyde, and blocked using 10% DS, 1% BSA in PBS-T before incubation with the primary antibody (Iba1, CD11b, CX3CR1, CD68, CD31, CD206, or NeuN). The specific antibody working dilutions are given in the resources table (Supplementary Table 2).

Terminal deoxynucleotidyl transferase dUTP nick end labeling (TUNEL, In Situ Cell Death Detection Kit, Merck Group, Darmstadt, Germany) staining was used according to the manufacturer guidance in order to detect cell death rates, and 4′,6-diamidino-2-phenylindole (DAPI) staining was used in order to stain nuclei. Five pictures per hemisphere were taken in the striatum as region of interest (ROI) and five pictures per well of cells were taken with the Axioplan 2 fluorescence microscope (Carl Zeiss, Jena, Germany). The software ImageJ (National Institutes of Health, USA) was used for cell counting and intensity quantification. The negative and unstained controls of staining were presented in the Supplementary Materials.

### Flow cytometry analysis

Infiltrating microglia and the subset of M2 polarization microglia in cerebral hemispheres were determined by flow cytometry with a fluorescence-activated cell sorter as described [64, 65]. Briefly, the ischemic hemispheres were mechanically homogenized in lysis buffer (0.5% BSA, 5% glucose, 10 mg/ml DNase, PBS) and centrifuged at 1600 rpm for 10 min. Thereafter, the pellet was solved in 30% Percoll solution (GE Healthcare, USA) and loaded on the Percoll gradient containing 45% and 70% Percoll phases. Following centrifugation, the immune cells between the phases were aspirated and solved in working solution (3% fetal bovine serum in PBS). Before antibody labeling, the cell suspension was incubated at 4 °C for 10 min with anti-mouse Fc-Block (final concentration of 2.5 μg/ml) to prevent unspecific binding. After washing, the cells were incubated with anti-CD45, anti-CD11b, and anti-CD206 (BioLegend, San Diego, USA) overnight. Flow cytometry quantification was obtained with the software FlowJo v. 10.5.3 (BD FACSDiva™). Gating strategy of M2 microglia was presented in Supplementary Fig. III B. Likewise, the representative 2D plots of flow cytometry can be found in the Supplementary Materials.

### Analysis of poststroke motor coordination deficits

The mice were trained on days 1 and 2 before the induction of stroke to ensure proper test behavior. The tests for analysis of motor coordination were performed at the time points (prestroke day 1 and poststroke days 2, 5, and 7) given using the rotarod test, the tightrope test, the balance beam test, the corner turn test, and paw slips recording, as previously described [66–68]. The rotarod, the balance beam, and the tightrope tests were performed three times on each test day and the mean values calculated. For the rotarod test, the readout parameter was the time until the mice dropped off, with a maximal testing time of 300 s. The parameter of the balance beam test was the time until the mice reached the platform, with a maximal testing time of 60 s. Meanwhile, the number of paw slips was documented and averaged out of three trials for each mouse. Assessment of the tightrope test results was done using a validated score ranging from 0 (minimum) to 20 (maximum). The details of the tightrope test score sheet can be found in Supplementary Table 3. The corner turn test included ten trials per test day during which the laterality index (number of right turns per 10 trials) was calculated. High laterality indexes approximating a score of 1 indicate severe motor coordination impairment.

### Statistical analysis

Results are shown as means ± standard deviation (SD). Statistical analysis was performed using the Student’s *t* test to compare two groups. The statistical significance of differences between several groups was assessed by a one-way analysis of variance (ANOVA) and a two-way ANOVA for factorial comparisons and by Bonferroni or Tukey−Kramer’s test for multiple comparisons. Differences were considered significant when *p* values were <0.05 using GraphPad Prism 6.0 (GraphPad, San Diego, CA, USA).

## Supplementary information

Supplemental data

## Data Availability

All data generated or analyzed during this study are included in this published article and its supplementary information files.
