# Supplementary material for: Extracellular vesicles from hypoxia-preconditioned microglia promote angiogenesis and repress apoptosis in stroke mice via the TGF-β/Smad2/3 pathway
Source: Cell Death Dis. 2021 Nov 9;12(11):1068. doi: 10.1038/s41419-021-04363-7 (PMC8578653; doi:10.1038/s41419-021-04363-7)
Supplement: Supplementary file 1 — Supplemental data [file 41419_2021_4363_MOESM1_ESM.pdf]

SUPPLEMENTARY MATERIALS

Extracellular vesicles from hypoxia-preconditioned microglia promote angiogenesis and repress apoptosis in stroke mice via the TGF-β/Smad2/3 pathway

Lin Zhang[1], Wei Wei[1], Xiaoyu Ai[1], Ertugrul Kilic[2], Dirk M Hermann[3], Vivek Venkataramani[4,5], Mathias Bähr[1], and Thorsten R Doeppner[1,2]

[1]University Medical Center Göttingen, Department of Neurology, Göttingen, Germany

[2]Istanbul Medipol University, Regenerative and Restorative Medical Research Center, Istanbul, Turkey

[3]University Hospital Essen, University of Duisburg-Essen, Department of Neurology, Essen, Germany

[4]University Hospital Frankfurt, Department of Medicine II, Frankfurt, Germany

[5]University Medical Center Göttingen, Institute of Pathology, Göttingen, Germany

Correspondence: Thorsten R. Doeppner, MD-M.Sc.
Department of Neurology
University Medical Center Göttingen
Robert-Koch-Str. 40, 37075 Göttingen, Germany
Phone: +49-551-3966660, fax: +49-551-399348
email: thorsten.doeppner@med.uni-goettingen.de

Supplementary Materials and Methods

Characterization of EVs

Transmission electron microscopy (TEM) was used to investigate the microstructure of EVs from OGD-preconditioned microglia or TGF-β1 siRNA-transfected microglia. Briefly, formvar-coated TEM grids (copper, 150 hexagonal mesh, Science Services, Munich, Germany) were put on the top of a droplet of the respective EV fraction and incubated for 10 min. Then, the grids were washed and incubated with ultrapure water. For contrast, the grids were incubated for 5 min on droplets of uranylacetate-oxalate, followed by a 5-min incubation on droplets of a 1:9 dilution of 4 % uranylacetate in 2 % methylcellulose. After draining the methylcellulose from the grids using a filter paper and drying of the methylcellulose film as previously described, samples were imaged with a LEO912 transmission electron microscope (Carl Zeiss Microscopy, Oberkochen, Germany) and images were taken using an onaxis 2k CCD camera (TRS-STAR, Stutensee, Germany).

The characterization of EVs was done by measuring the expression of the specific markers Alix and Tsg101 as well as by Western blot analysis of the EV-associated protein markers CD63, CD81 and CD9. For both size determination and quantification analysis of enriched EVs, a **nanoparticle tracking analysis (NTA)** was performed using the Nanosight platform (NanoSight LM10, Malvern Panalytical, Kassel, Germany). As shown previously [1], 1:1,000 PBS-diluted samples were measured in duplicate, and 400 µl of the diluted sample were injected into the measurement chamber. Each sample was measured three times, and the length of the video of each measurement was set to 30 s.

Reference

1. Sokolova V, Ludwig AK, Hornung S, Rotan O, Horn PA, Epple M and Giebel B. Characterisation of exosomes derived from human cells by nanoparticle tracking analysis and scanning electron microscopy. *Colloids Surf B Biointerfaces*. 2011;87:146-50.

Supplementary Figure I

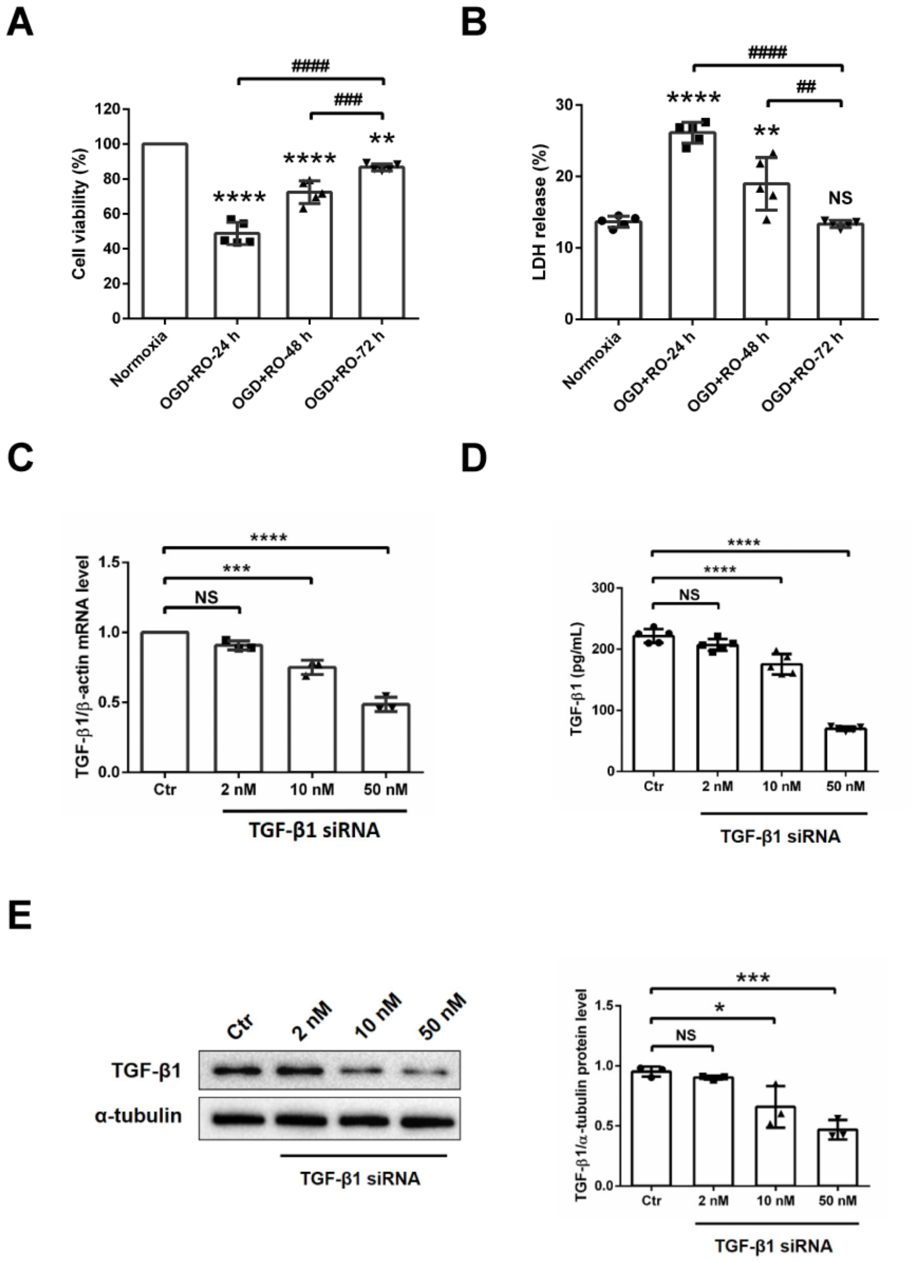

S-Figure I. (A) Cell viability was analyzed in primary microglia exposed to 4 h of oxygen-glucose deprivation (OGD) followed by different time of reoxygenation (RO) using the Thiazolyl Blue Tetrazolium Bromide (MTT) assay in four groups: normoxia, OGD with 24 h RO, OGD with 48 h RO and OGD with 72 h RO (n = 5). Cells incubated in the normoxia control group were defined as 100 % cell survival. (B) OGD-induced cell toxicity of primary microglia was further assessed in the lactate dehydrogenase (LDH) release assay (n = 5) in the aforementioned groups. (C) Quantitative analysis of

TGF-β1 mRNA expression in primary microglia using quantitative real-time polymerase chain reaction (qRT-PCR) normalized with the housekeeping gene β-actin ($n = 3$) in different concentrations of TGF-β1 siRNA groups (2 nM, 10 nM, and 50 nM). The control group was applied with negative control siRNA. (D) For cytokine quantification in culture supernatants, we measured the production of TGF-β1 by using Enzyme-linked immunosorbent assay (ELISA) in the same four groups. The TGF beta-1 Human/Mouse Uncoated ELISA Kit (Thermo Fisher Scientific, Waltham, Massachusetts, USA) was applied according to the manufacturer's instructions ($n = 5$). (E) Quantitative analysis of TGF-β1 expression in the above four groups by Western blot analysis normalized with the housekeeping protein α-tubulin ($n = 3$). Data are expressed as mean ± SD. NS: no significance, **$p < 0.01$, ***$p < 0.001$, ****$p < 0.0001$, ##$p < 0.01$, ###$p < 0.001$, and ####$p < 0.0001$. Abbreviations: EVs, extracellular vesicles; OGD, oxygen-glucose deprivation; RO, reoxygenation; MTT, thiazolyl blue tetrazolium bromide; LDH, lactate dehydrogenase; qRT-PCR, quantitative real-time polymerase chain reaction; ELISA, Enzyme-linked immunosorbent assay.

Supplementary Figure II

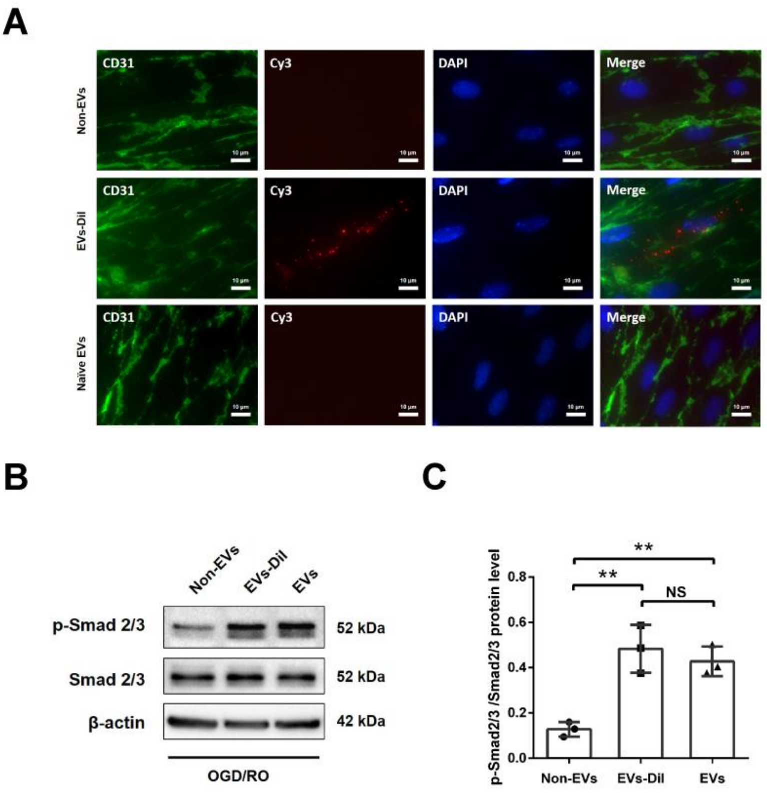

D

Groups	1	2	3	4	5	6
OGD	-	+	+	+	+	+
EVs	-	-	+	+	-	-
TGF-β1R inh	-	-	-	+	-	-
si-EVs	-	-	-	-	+	+
rTGF-β1	-	-	-	-	-	+

OGD: the cells were exposed to oxygen-glucose deprivation (OGD) and 24 h reoxygenation (RO);
EVs: 1 μg/ml EVs derived from the microglia preconditioned with OGD/RO;
TGF-β1R inh: 2 μM TGF-β1 receptor inhibitor;
si-EVs: 1 μg/ml EVs derived from microglia preconditioned with OGD/RO and TGF-β1 siRNA (50 nM);
rTGF-β1: 10 ng/ml recombinant TGF-β1

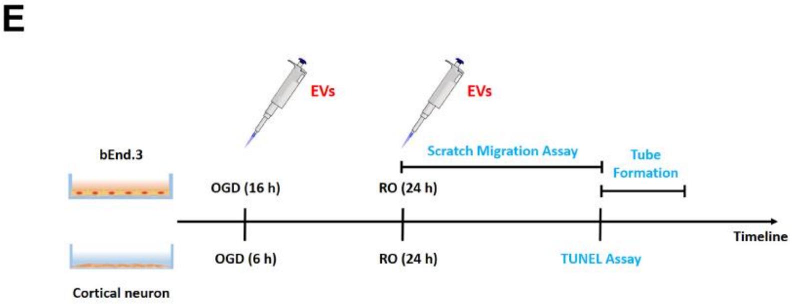

S-Figure II. (A) EVs labeled with DiI (red) were taken up into the cytoplasm of CD31+ (green) bEnd.3 endothelial cells in the EVs-DiI group. DiI labeling had no impact on endothelial cell morphology or functional state when compared to endothelial cells treated with naïve EVs. (B-C) Likewise, both EVs-DiI and naïve EVs activated the Smad2/3 signal pathway after OGD/RO in endothelial cells when compared to the non-EV group (n = 3). (D) To investigate the EV effect on the TGF-β/Smad2/3 pathway in bEnd.3 and primary cortical neurons, we developed six groups: group 1 (treatment with drug solvent in normoxic condition), group 2 (OGD/RO treatment with drug solvent), group 3 (1 ng/ml EV incubation during OGD/RO, EVs derived from OGD-preconditioned microglia), group 4 (2 μM TGF-β1R inhibitor treatment in group 3), group 5 (1 ng/ml si-EV incubation during OGD/RO, si-EVs derived from OGD-preconditioned and TGF-β1 siRNA transfected microglia), and group 6 (10 ng/ml recombinant TGF-β1 treatment in group 5). (E)The timeline of *in vitro* experiments including OGD, RO, EV incubations, and post-OGD assays (scratch migration assay, tube formation in bEnd.3, and TUNEL assay in cortical neurons). Data are expressed as mean ± SD. NS: no significance, $^{**}p < 0.01$. Abbreviations: EVs, extracellular vesicles; OGD, oxygen-glucose deprivation; RO, reoxygenation.

Supplementary Figure III

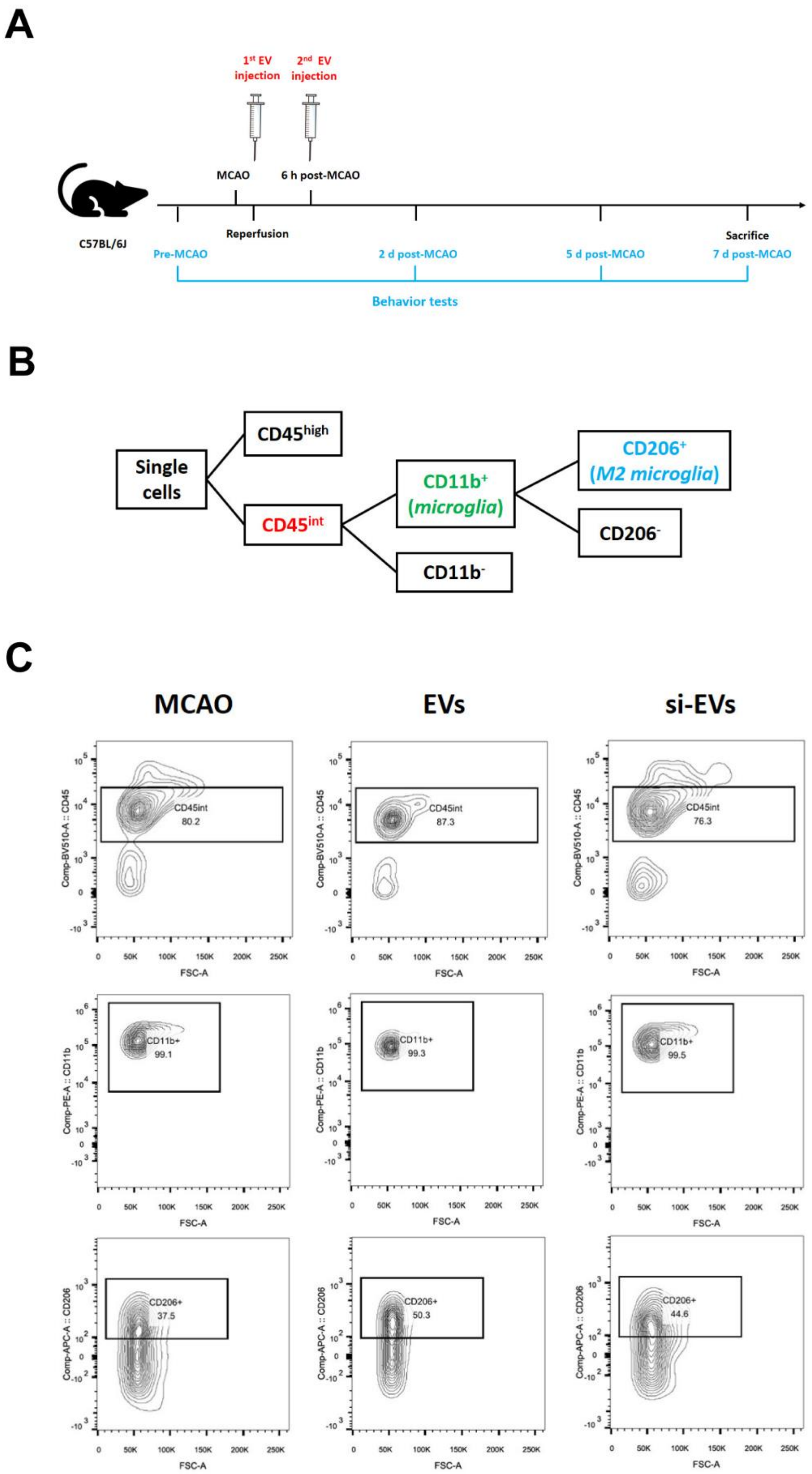

S-Figure III. (A) The timeline of *in vivo* experiments including middle cerebral artery occlusion (MCAO), EV treatment, and behavior tests. (B) Gating strategy for flow

cytometry analysis. The subset of M2 microglial cells (CD45^{int}CD11b+CD206+) was analyzed. (C) Representative flow cytometry measurement of the ischemic hemisphere in MCAO-exposed mice with vehicle PBS injection and MCAO-exposed mice treated with EVs or si-EVs. All groups were exposed to 60 min of MCAO followed by 7 days of survival. For analysis, the cells were stained with antibodies against CD45, CD11b and CD206. Abbreviations: EVs, extracellular vesicles; MCAO, middle cerebral artery occlusion.

Supplementary Table 1—Experimental groups and survival rates of mice.

	**Behavior tests and immunofluorescence**			**Western blot analysis**			**FACS**		
Groups	Number	Dead	Survival	Number	Dead	Survival	Number	Dead	Survival
MCAO	16	1	93.8 %	5	0	100 %	4	0	100 %
EVs	15	1	93.3 %	5	0	100 %	5	1	80 %
si-EVs	14	2	85.7 %	6	1	83.3 %	5	1	80 %

A total of 75 mice was used in the *in vivo* experiments including behavior tests, immunofluorescence staining, Western blot analysis, and FACS analysis. Two mice in the EV group and two mice in si-EV group died before the first EV injection because of severe strokes. Two mice in the si-EV group were sacrificed because of severe pain on the second day after surgery. One mouse in the MCAO group was excluded because of unsuccessful surgery. There was no animal dead in the remaining groups before reaching the end time points in question. Abbreviations: FACS, fluorescence-activated cell sorting; MCAO, middle cerebral artery occlusion; EVs, extracellular vesicles; si-EVs, extracellular vesicles derived from TGF-β1 siRNA-transfected microglia.

Supplementary Table 2—Major Resources

REAGENT/RESOURCE	SOURCE	IDENTIFIER	APPLICATION
Ⅰ. Antibodies			
Iba1	WAKO	011-27911	2 μg/ml (for IF)
CD11b	Abcam	ab75476	2 μg/ml (for IF)
CX3CR1	Thermo Fisher Scientific	PA5-19910	2 μg/ml (for IF)
CD68	BioRad	MCA341F	2 μg/ml (for IF)
TGF-β1	Abcam	ab92486	0.5 μg/ml (for WB)
GAPDH	GeneTex	GTX627408	0.1 μg/ml (for WB)
CD206	Abcam	ab64693	2 μg/ml (for IF)
β-actin	Abcam	ab6276	0.2 μg/ml (for WB)
Alix	BD Biosciences	611620	0.5 μg/ml (for WB)
CD63	Biorbyt	orb11317	0.5 μg/ml (for WB)
CD81	Abcam	ab155760	0.5 μg/ml (for WB)
CD9	Abcam	ab92726	0.5 μg/ml (for WB)
Tsg101	GeneTex	GTX70255	1 μg/ml (for WB)
CD31	Abcam	ab28364	5 μg/ml (for IF)
Smad 2/3	Abcam	ab202445	0.5 μg/ml (for WB)
p-Smad 2/3	Abcam	ab272332	0.5 μg/ml (for WB)
Bcl-2	Cell Signaling Technology	#3498	0.5 μg/ml (for WB)
Bax	Abcam	ab7977	0.5 μg/ml (for WB)
NeuN	Abcam	ab104225	2 μg/ml (for IF)
Brdu	Abcam	ab6326	2 μg/ml (for IF)
CD45	BD Biosciences	563891	20 μg/ml (for FACS)
CD206	BioLegend	141708	20 μg/ml (for FACS)
CD11b	BD Biosciences	552850	20 μg/ml (for FACS)
Ⅱ. Chemicals and Recombinant Proteins			
L-glutamine	Thermo Fisher Scientific	25030024	100 μl L-glutamine in 10 ml medium
TGF-β1 receptor inhibitor	Sigma-Aldrich	SB-525334	2 μM in DMSO
Recombinant human TGF-β1	PeproTech	100-21	100 μg TGF-β1 in 50ml PBS with 0.1% BSA
Antibiotic/antimycotic (100x)	Thermo Fisher Scientific	15240062	100 μl AA in 10 ml medium
Thiazolyl Blue Tetrazolium Bromide	Sigma-Aldrich	M5655	5 mg/ml in PBS
Matrigel	Corning	#354230	undiluted
Trypsin-EDTA 0.25%	Thermo Fisher	25200-056	undiluted

	**Scientific**		
**Poly-D-lysine**	**Sigma-Aldrich**	**P6407**	**5mg PDL in 50 ml H_2O (0.1 mg/mL)**
**Poly-L-ornithine**	**Sigma-Aldrich**	**P3655**	**5mg PLO in 50 ml H_2O (0.1 mg/mL)**
**Laminin**	**Sigma-Aldrich**	**L2020**	**2 µg/ml in PBS**
**Penicillin/streptomycin**	**Thermo Fisher Scientific**	**15140122**	**500 µl pen/strep in 50 ml medium**
**B27**	**Thermo Fisher Scientific**	**17504001**	**1ml B27 in 50 ml medium**
**Polyethylene glycol 6000**	**Sigma-Aldrich**	**8074911000**	**20g Peg6000 in 200 ml H_2O with 75 mM NaCl**
**DiI**	**Thermo Fisher Scientific**	**D3911**	**10 µM in DMSO**
**DAPI**	**AppliChem**	**A1001**	**1 µg/ml in PBS/TBS**

WB: Western blot

IF: Immunofluorescence staining

FACS: Fluorescence-activated Cell Sorting

Supplementary Table 3—Score sheet for tightrope test

Score	Time (s)	Platform arrival
20	1-6	+
19	7-12	+
18	13-18	+
17	19-24	+
16	25-30	+
15	31-36	+
14	37-42	+
13	43-48	+
12	49-54	+
11	55-60	+
10	55-60	−
9	49-54	−
8	43-48	−
7	37-42	−
6	31-36	−
5	25-30	−
4	19-24	−
3	13-18	−
2	7-12	−
1	1-6	−
0	0	−

The tightrope test was performed three times on each test day, and the mean values were calculated. The results were assessed according to both time on the rope (in seconds) and platform arrival ("+" for arrival and "−" for non-arrival). The scores ranged from 0 (minimum) to 20 (maximum).

Supplementary full scans of Western blots

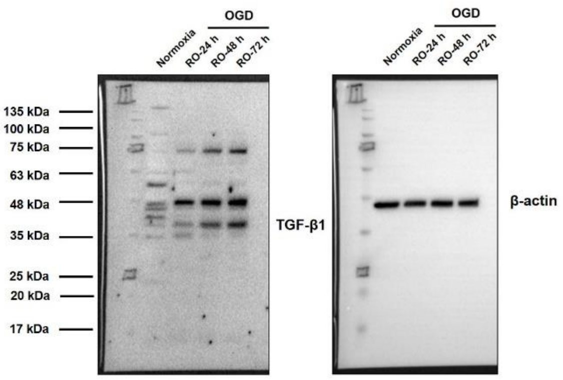

Full scans of Western blots shown in Fig. 1B. TGF-β1 and β-actin.

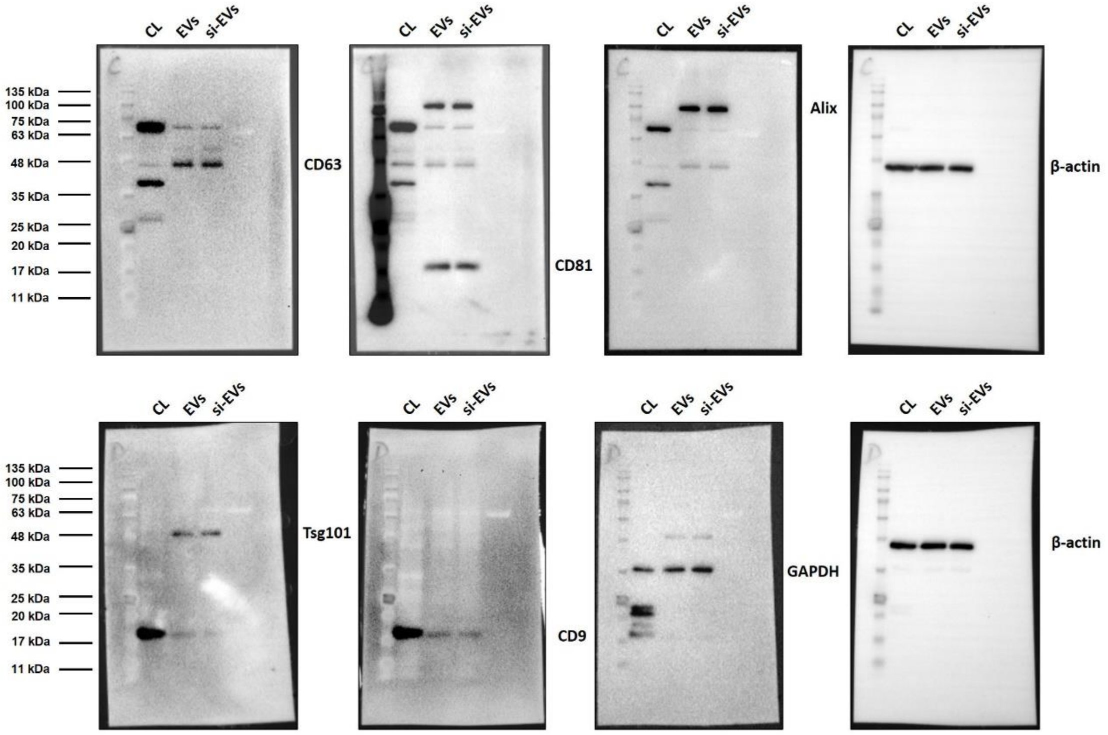

Full scans of Western blots shown in Fig. 2B. CD63, CD81, Alix, Tsg101, CD9, GAPDH and β-actin.

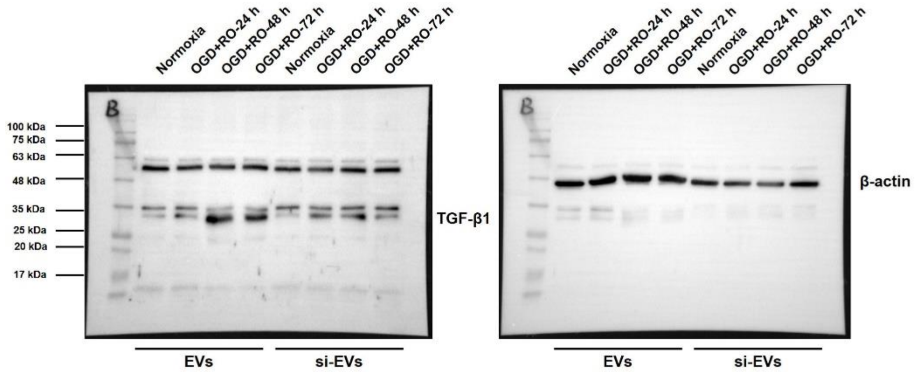

Full scans of Western blots shown in Fig. 2E. TGF-β1 and β-actin.

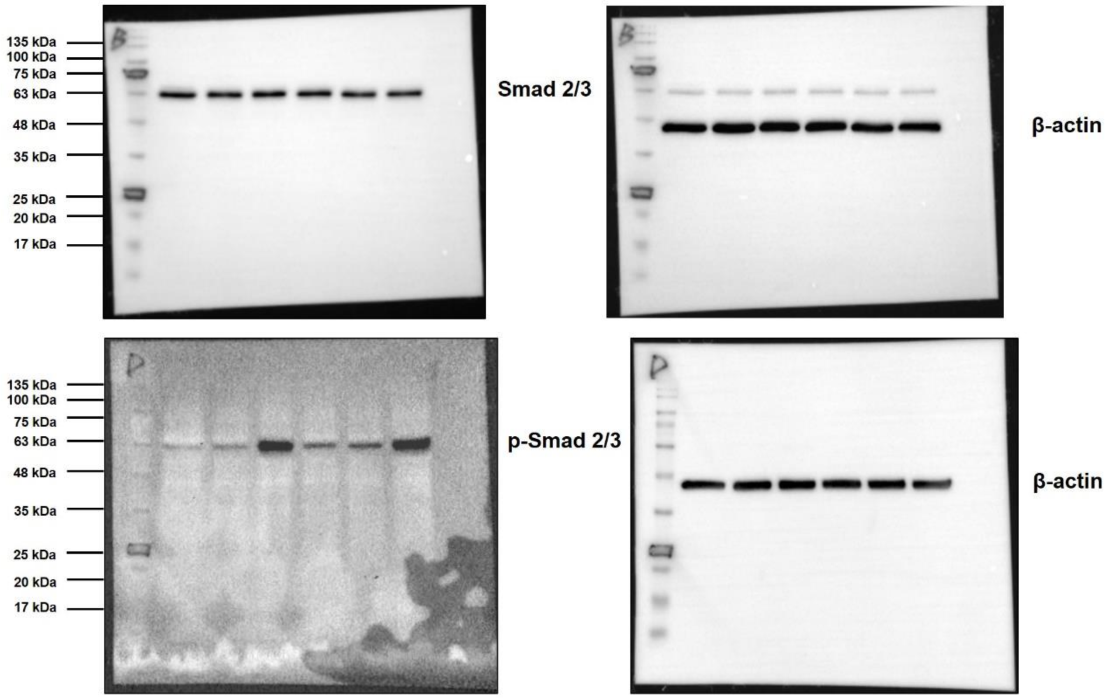

Full scans of Western blots shown in Fig. 3B. Smad 2/3, p-Smad 2/3 and β-actin.

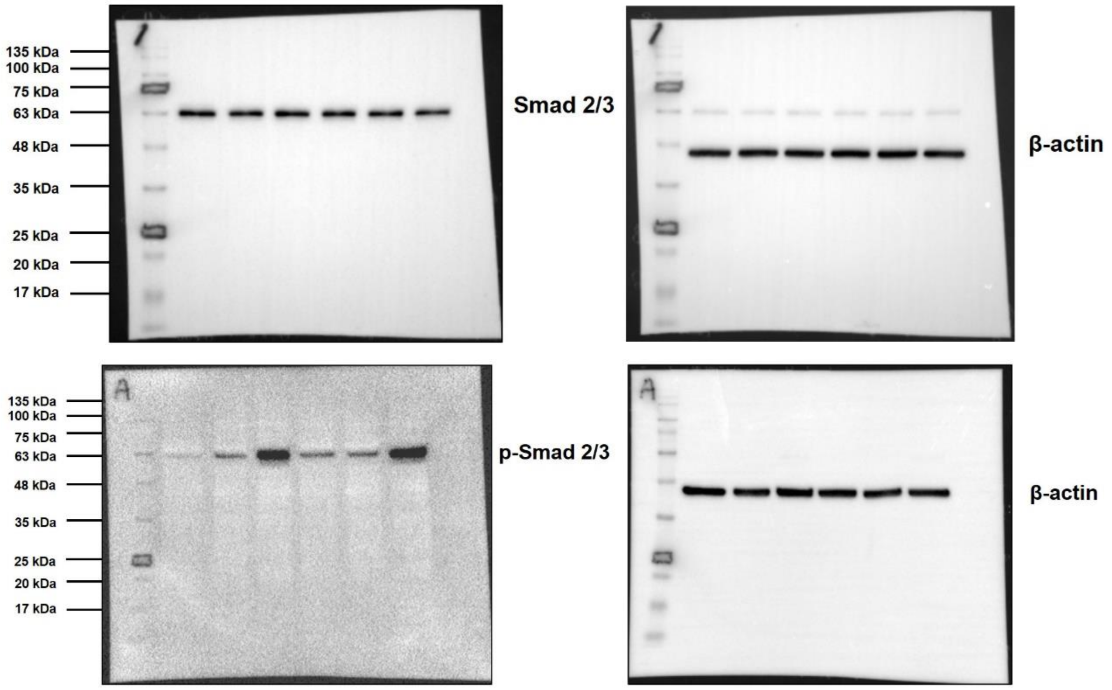

Full scans of Western blots shown in Fig. 4B. Smad 2/3, p-Smad 2/3 and β-actin.

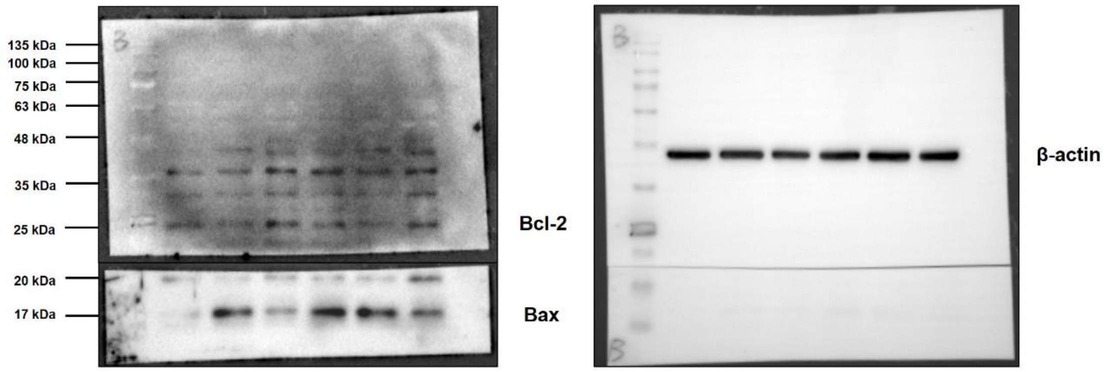

Full scans of Western blots shown in Fig. 4C. Bcl-2, Bax and β-actin.

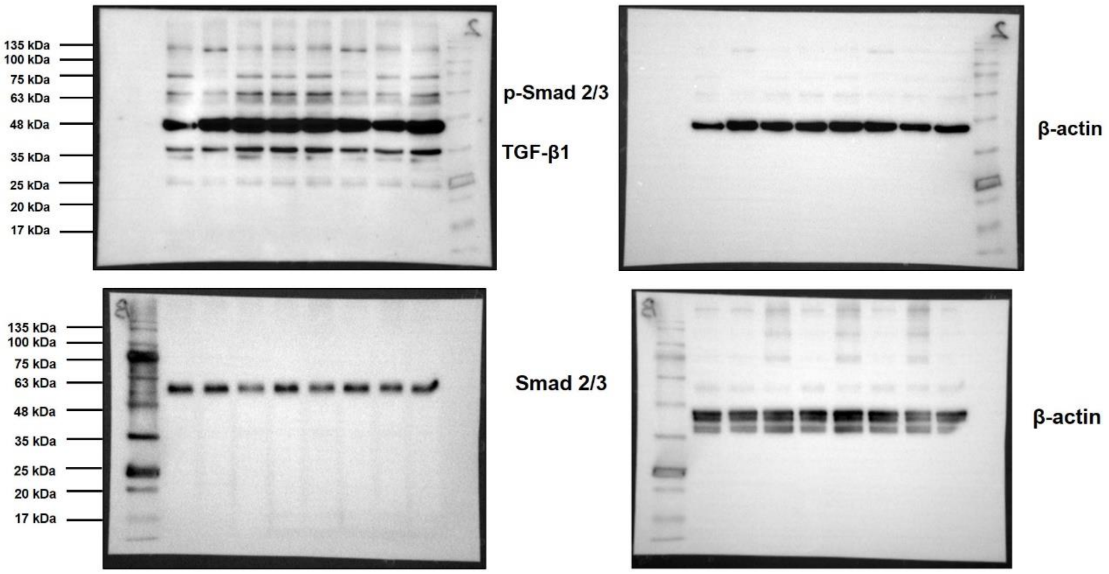

Full scans of Western blots shown in Fig. 5A. TGF-β1, Smad 2/3, p-Smad 2/3 and β-actin.

Supplementary 2D plots of FACS

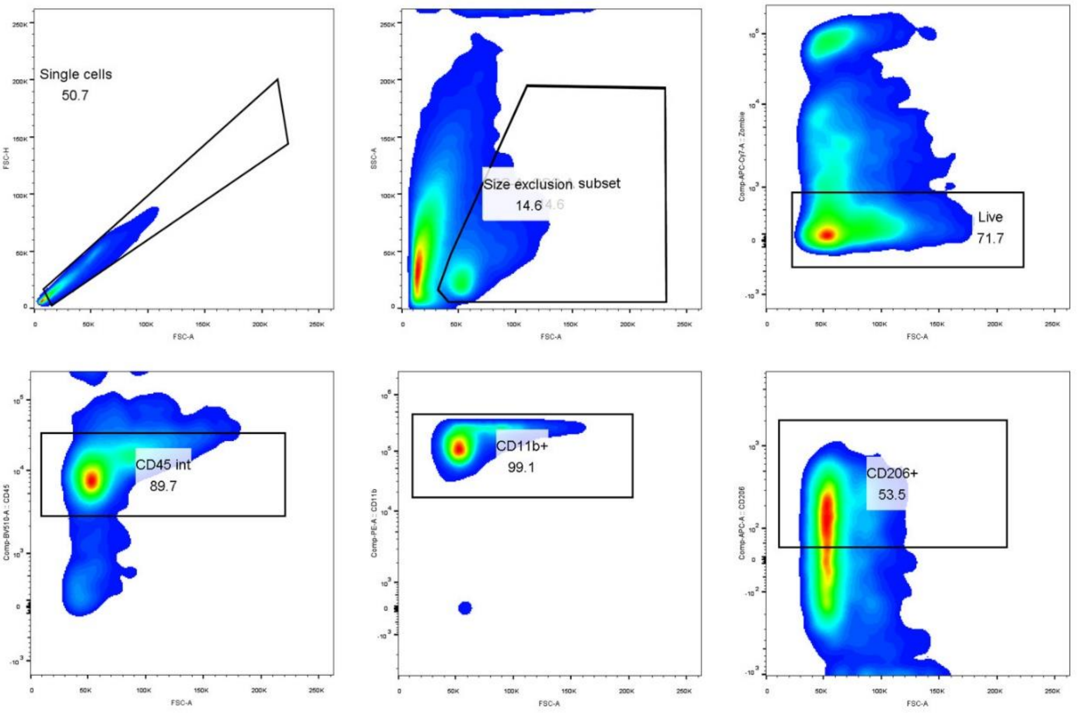

According to the Gating strategy for flow cytometry analysis in **S-Figure III**, we analyzed ratio of M2 microglial cells (CD45intCD11b+CD206+) in each ischemic brain by the software FlowJo v. 10.5.3 (BD FACSDiva™).

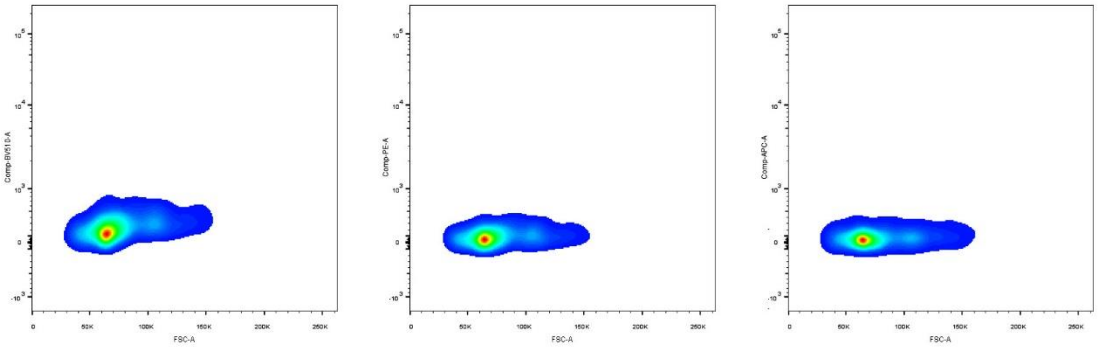

To determine the background autofluorescence to set the negative population allowing cells stained with CD45, CD11b, and CD206 to be visualized, we perform the unstained controls above.

Supplementary negative and unstained controls of staining

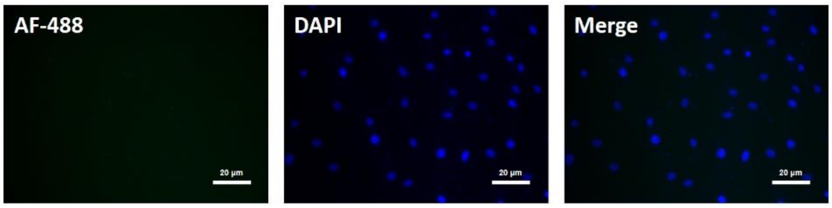

As a **negative control**, the secondary antibody AlexaFlour488 donkey anti-mouse IgG (1:250) was incubated with microglia, and there is no unspecific staining on the slides.

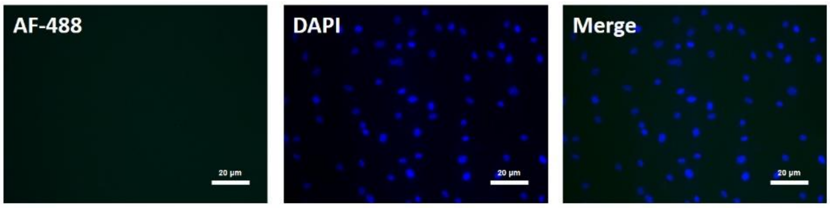

As a **negative control**, the secondary antibody AlexaFlour488 donkey anti-rabbit IgG (1:250) was incubated with bEnd.3, and there is no unspecific staining on the slides.

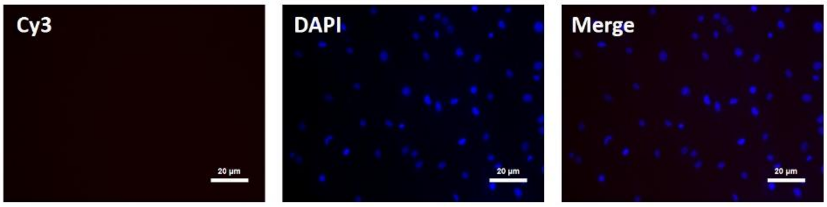

As a **negative control**, the secondary antibody Cy3 donkey anti-rabbit IgG (1:250) was incubated with microglia, and there is no unspecific staining on the slides.

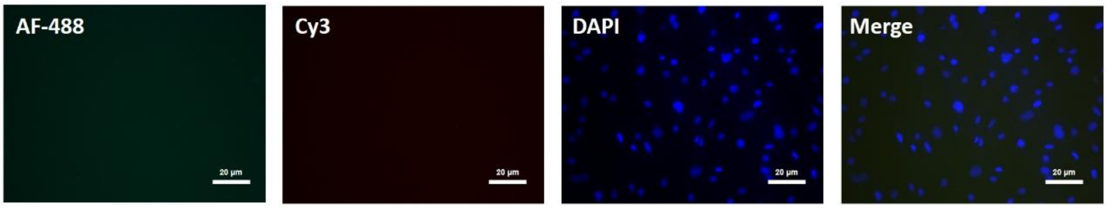

As an **unstained control**, only DAPI was detected with the microglia, in which does not yield any unspecific signal.

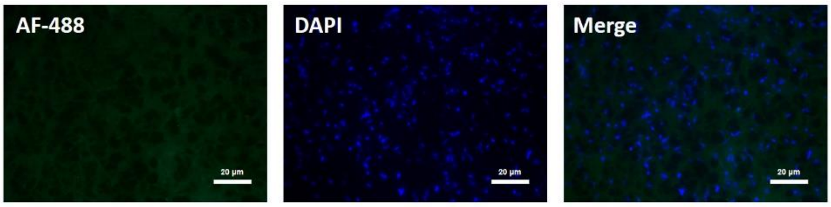

As a **negative control of staining**, the secondary antibody AlexaFlour488 donkey anti-rabbit IgG (1:250) was incubated with tissue slides, which does not yield significant unspecific staining in these sections.

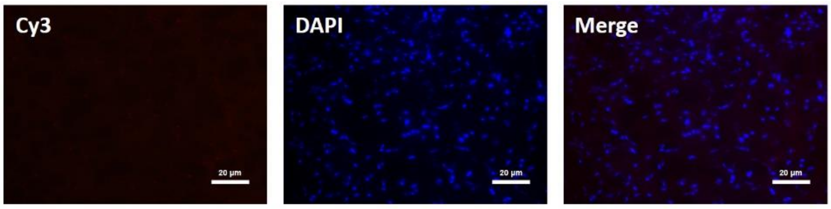

As a **negative control of staining**, the secondary antibody Cy3 donkey anti-rat IgG (1:250) was incubated with tissue slides, which does not yield significant unspecific staining in these sections.

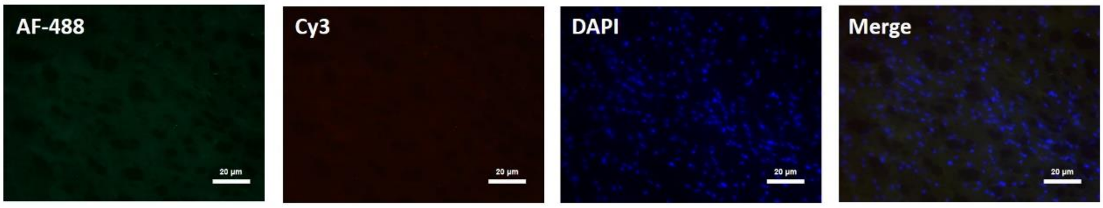

As an **unstained control**, only DAPI was detected with the tissue slides, which does not yield significant unspecific staining in these sections.
